# Nanosuspension Innovations: Expanding Horizons in Drug Delivery Techniques

**DOI:** 10.3390/pharmaceutics17010136

**Published:** 2025-01-19

**Authors:** Shery Jacob, Fathima Sheik Kather, Sai H. S. Boddu, Mahesh Attimarad, Anroop B. Nair

**Affiliations:** 1 Department of Pharmaceutical Sciences, College of Pharmacy, Gulf Medical University, Ajman 4184, United Arab Emirates; fathima.sheik@gmu.ac.ae; 2 Department of Pharmaceutical Sciences, College of Pharmacy and Health Sciences, Ajman University, Ajman P.O. Box 346, United Arab Emirates; s.boddu@ajman.ac.ae; 3 Center of Medical and Bio-Allied Health Sciences Research, Ajman University, Ajman P.O. Box 346, United Arab Emirates; 4 Department of Pharmaceutical Sciences, College of Clinical Pharmacy, King Faisal University, Al-Ahsa 31982, Saudi Arabia; mattimarad@kfu.edu.sa (M.A.); anair@kfu.edu.sa (A.B.N.)

**Keywords:** nanosuspension, nanocrystal, solid dispersion, preparation methods, stabilizers, drug delivery systems, in vitro characterization, patents, marketed formulations, clinical trials

## Abstract

Nanosuspensions (NS), with their submicron particle sizes and unique physicochemical properties, provide a versatile solution for enhancing the administration of medications that are not highly soluble in water or lipids. This review highlights recent advancements, future prospects, and challenges in NS-based drug delivery, particularly for oral, ocular, transdermal, pulmonary, and parenteral routes. The conversion of oral NS into powders, pellets, granules, tablets, and capsules, and their incorporation into film dosage forms to address stability concerns is thoroughly reviewed. This article summarizes key stabilizers, polymers, surfactants, and excipients used in NS formulations, along with ongoing clinical trials and recent patents. Furthermore, a comprehensive analysis of various methods for NS preparation is provided. This article also explores various in vitro and in vivo characterization techniques, as well as scale-down technologies and bottom-up methods for NS preparation. Selected examples of commercial NS drug products are discussed. Rapid advances in the field of NS could resolve issues related to permeability-limited absorption and hepatic first-pass metabolism, offering promise for medications based on proteins and peptides. The evolution of novel stabilizers is essential to overcome the current limitations in NS formulations, enhancing their stability, bioavailability, targeting ability, and safety profile, which ultimately accelerates their clinical application and commercialization.

## 1. Introduction

Advancements in drug discovery technologies, such as combinatorial chemistry, fragment-based drug discovery, natural product screening, virtual screening, genomics and proteomics approaches, phenotypic screening, computational de novo drug designs, cheminformatics-based approaches, bioinformatics and systems biology, and high throughput screening—including artificial intelligence and machine learning—have led to the generation of numerous potential drug candidates with excellent target receptor binding [1]. On the other hand, the challenging physicochemical properties of drugs like high lipophilicity, a large molecular size, stable crystal lattices, and insufficient hydrogen bonding render them poorly soluble in water, leading to their reduced and variable bioavailability. The surface modification of these entities is often problematic due to their high hydrophobicity, complex structures, and stability issues, which restrict advancements into an effective formulation [2]. It is widely recognized that particle size reduction increases the surface area, which can greatly improve the dissolution rate and bioavailability, as indicated by the classical Noyes–Whitney equation [3]. Pharmaceutical nanosuspensions (NS) are nanodispersed solid–liquid (S/L) systems consisting of nanoscale insoluble drug particles in a solid-phase state, dispersed mainly in an aqueous medium and typically stabilized by surfactants or polymers to prevent particle aggregation or sedimentation. The stabilizing mechanisms rely on the emergence of physical and/or electrostatic barriers between particles to counteract attractive forces, such as Van der Waals interactions. The crystalline state of drug particles significantly influences the physicochemical stability and pharmacokinetic behavior of NS. However, the drug particles may also exist in a partially amorphous state and exhibit unique behaviors, including enhanced solubility, but potentially reduced stability compared to fully crystalline forms [4]. The NS technique is one of the feasible options available when a therapeutic molecule faces numerous limitations, such as high dose requirements, low intrinsic aqueous solubility, an inability to form salts, a large molecular weight, high log P, and high melting point. NS enhances bioavailability and solubility by reducing the drug particle size to the nanometer range, which increases the surface area and improves the dissolution rates. This approach is particularly effective for Biopharmaceutics Classification System (BCS) class II and IV drugs, which are characterized by poor solubility. For BCS class II drugs such as griseofulvin, NS enhance dissolution-limited absorption by providing a greater surface area for their interaction with the gastrointestinal fluids. According to the Developability Classification System, they are particularly beneficial for class IIa drugs, where absorption is primarily constrained by the dissolution rate [5]. NS stabilizes the drug in a supersaturated state and prevents precipitation, ensuring consistent bioavailability. This method is particularly beneficial for drugs with pH-dependent solubility, such as ibuprofen and ketoprofen, as it maintains solubility across varying gastrointestinal environments. Nanosized cocrystals have emerged as an effective approach to overcome the low water solubility of BCS class II/IV drugs by utilizing co-crystallization and nanocrystallization techniques. Various bottom-up and top-down preparation methods have been employed to produce cocrystals, using appropriate stabilizers and co-formers, while applying Quality by Design (QbD) and Process Analytical Technology. These nanocrystalline structures, consisting of two or more components arranged in a crystal lattice with a defined stoichiometric ratio and assembled via hydrogen bonds or other noncovalent interactions, have been shown to enhance the long-term stability and dissolution rates. Carbamazepine–nicotinamide, 4-aminosalicylic acid–sulfamethazine, and lamivudine–zidovudine nanococrystals have demonstrated significant improvements in their solubility and stability [6,7,8]. Different co-crystallization approaches, including solvent-assisted grinding and surfactant-assisted grinding, have been utilized to prepare nanocrystalline ketoprofen and naproxen, respectively [9,10]. Moreover, the solubility, dissolution rate, and absorption of many low-aqueous-soluble phytochemicals, such as quercetin, curcumin, baicalein, piperine, lutein, and betulinic acid have been found to increase when formulated into nanocrystals (NCs) using various co-formers [11,12,13,14,15,16]. In the case of the BCS class IV drugs, such as aprepitant, while their solubility improves, the absorption is often limited by permeability. NS can still enhance the local concentration gradient, indirectly improving the permeability in some cases.

NS provides numerous advantages, including improved oral bioavailability by enhancing the maximum solubility and dissolution rate of the active ingredients, which further enhances cell-mediated endocytosis [17]. Additionally, NS reduces inter-subject variability and minimizes the differences that occur between fasting and fed states [18]. With a simple composition and minimal use of excipients, NS are easy to prepare, scale-up, and produce rapidly and cost effectively [19]. By allowing a large drug amount within a minimal dose volume, NS are particularly beneficial for parenteral and ophthalmic drug delivery systems, as they reduce the need for excessive nonaqueous solvents and extreme pH conditions [20]. For instance, cyclodextrin’s inherent large molecular weight significantly increases the bulk of pharmaceutical formulations when used for molecular complexation [21]. NS address unique drug delivery challenges related to active pharmaceutical ingredients by maintaining them in a crystalline state and allowing for increased drug loading (50–100%) during formulation preparation. The nanometer size of the particles improves passive targeting, allowing them to navigate and accumulate more effectively in specific tissues or cells [22]. Solidified NS offer enhanced physical stability and can be developed into solid dosage forms like tablets, capsules, films, pellets, powders, or granules [17]. Moreover, NS can be adapted for various administration routes, including parenteral, pulmonary, topical, and ophthalmic, and can be sterilized using conventional methods like filtration, dry heat, steam, and radiation. Additional advantages include greater stability, sustained drug release, improved efficacy by tissue targeting, a minimized first-pass effect, and profound lung deposition [23].

Achieving and maintaining the desired nanoscale particle size is a key factor for the bioavailability and therapeutic performance of the drug. NS are prone to instability, including particle aggregation, sedimentation, and changes in the crystalline structure; therefore, they require suitable stabilizers. The stability of nanometer-sized particles in NS is primarily due to their consistent particle size, obtained via different manufacturing techniques. For NS to avoid spontaneous crystal formation due to the Ostwald ripening process, their particle size must be constant throughout their shelf life [24]. Scaling up the production of NS from a laboratory to industrial scale while maintaining a consistent particle size, stability, and reproducibility requires sophisticated equipment and stringent quality control measures. Despite the complexities involved in manufacturing, choosing the correct unit operations, instruments, and process optimization could significantly mitigate these challenges. Meeting regulatory requirements for NS involves extensive characterization, safety testing, and validation. Addressing these challenges requires a multidisciplinary approach, integrating expertise in nanotechnology, pharmaceutical sciences, and regulatory affairs to achieve successful NS formulations.

The present review distinguishes itself by offering a comprehensive analysis of NS on diverse drug delivery routes, including oral, ocular, transdermal, pulmonary, and parenteral, compared to existing reviews [25,26,27,28,29]. It thoroughly examines the conversion and stabilization of oral NS into various dosage forms and explores advancements in stabilizers, polymers, surfactants, and excipients, highlighting their roles in enhancing stability, bioavailability, and targeting. Additionally, the current review extensively covers both scale-up and bottom-up preparation methods, emphasizing innovative techniques like sonocrystallization and supercritical fluid (SCF) methods. This work provides a broader and more detailed perspective on the advancements, challenges, and future potential of NS.

## 2. Fundamental Principle

Due to their smaller size, nanoparticles (NPs) have a significantly increased surface area, which enhances their saturation solubility, as explained by the classical Ostwald–Freundlich equation and the Gibbs–Thomson effect [30]. Nanosizing also increases the solubility by forming high-surface-energy surfaces, disrupting the crystal lattice, and exposing hydrophobic surfaces to the aqueous medium. This increased solubility can promote the Ostwald ripening process, where tiny particles dissolve and transfer onto bigger particles, leading to particle growth and instability. Researchers used the time-dependent changes in the surface area during the dissolution process to describe the dissolution of a single particle by extending the Nernst–Brunner idea [31]. Interfacial energy has been found to have a considerable influence on the saturation solubility of the drug’s various polymorphic forms. This effect is particularly seen in metastable NS, which have higher surface energy and solubility compared to more stable coarse suspensions [32]. The dissolution rate of solid drugs is directly proportional to the available surface area, explained by the Nernst–Brunner/Noyes–Whitney equation [3]. According to the formula ΔG = γ_S/L_ΔA, where ΔA is the change in the surface area and γ_S/L_ is the interfacial tension between the solid particle and the aqueous medium, the change in surface free energy (ΔG) is determined by these factors. A system is considered thermodynamically stable when its total free energy is at a minimum (typically ΔG = 0). However, for dispersed systems such as emulsions, NS, and nanoemulsions, thermodynamic stability is rare. These systems often exhibit kinetic stability, meaning they remain stable over a certain timescale but are not in a true equilibrium state. Over time, such systems may phase-separate to minimize their total free energy, typically by reducing their interfacial area. For example, in NS, particle aggregation reduces the total surface area and, consequently, the surface free energy. This behavior underscores the importance of adequate stabilization mechanisms to maintain the dispersion stability. By reducing the γ_S/L,_ surfactants lower the surface free energy and stop particles from aggregating. Ionic surfactants impart a charge to the surface of the NPs, creating repulsive electrostatic forces between similarly charged particles [33]. A higher zeta potential (ZP) signifies better stability, as it implies stronger repulsive forces that inhibit aggregation. Polymers adsorb onto the surface of NPs, creating a physical barrier that could block the particles from growing larger and thus help in maintaining the nanoscale size. Moreover, polymers increase the viscosity of the suspension medium, reducing the mobility of particles and thus the likelihood of collisions leading to aggregation. A combination of nonionic polymers and ionic surfactants allows for the better surface coverage of the NPs compared to a single surfactant. This comprehensive stabilization approach ensures that the particles remain uniformly dispersed, preserving the enhanced dissolution and bioavailability benefits of the NS. The small size of NPs in a well-stabilized NS ensures that they remain dispersed due to Brownian motion, further enhancing the physical stability of the suspension.

## 3. Formulation Composition

The formation and stabilization of drug NCs are affected by various physical and chemical characteristics of the drugs, such as polymorphism, log P, enthalpy, and cohesive energy. Crystalline forms of NCs are generally more stable than amorphous forms, which are prone to instability [34]. Strongly hydrophobic drugs benefit from easier stabilizer surface coverage compared to hydrophilic drugs. Drugs with low enthalpy are susceptible to aggregation while being stored due to structural transitions in water, leading to instability. NS formulations commonly include stabilizers, polymers, surfactants, cryoprotectants, and other excipients to ensure stability and effectiveness. Key factors in stabilizing NCs include surface hydrophobicity, cohesion, and the selection of appropriate stabilizers. It is important to note that the activation energy of the NS system significantly influences particle aggregation and precipitation. Employing suitable stabilizers can raise the activation energy, thereby preventing crystal growth caused by Ostwald ripening [35]. Stabilizers enhance stability, typically through steric or electrostatic mechanisms, affected by factors such as ZP, pH, and the physicochemical properties of both the stabilizers and the drug [36]. Ionic surfactants stabilize NCs by forming an electric double layer, while polymers and nonionic surfactants provide spatial stability through adsorbed hydrophobic molecules and extended hydrophilic chains that maintain particle spacing [37]. Electrostatic stabilization is sensitive to electrolytes or acidic conditions, whereas steric stabilization is more robust but requires the careful selection of polymers based on the drug’s properties. Combining stabilizers with different mechanisms has been shown to produce synergistic effects, improving stability [38]. High-molecular-weight polymer stabilizers outperform low-molecular-weight ones by inducing spatial repulsion to counteract van der Waals forces and prevent particle aggregation [39]. These insights underscore the importance of tailored stabilizer selection and stabilization strategies to achieve a stable NS system. The stabilizer selection and concentration significantly impact the physical stability as well as in vivo efficiency of NS, with commonly used types including cellulose or their derivatives, phospholipids, pluronics, and nonionic surfactants. Selection relies on the drug’s physico-chemical properties, desired delivery route, and formulation objectives.

Poloxamers, amino-acid-based stabilizers, cellulose derivatives, and D-alpha-tocopheryl polyethylene glycol (PEG) 1000 succinate (TPGS) are widely used excipients in NS formulations, each contributing to stability and functionality. Poloxamer, a nonionic triblock copolymer consisting of poly(ethylene oxide)-poly(propylene oxide)-poly(ethylene oxide) blocks, is widely used in NS formulations as a stabilizer and surfactant [40]. Its amphiphilic nature allows it to adsorb onto the surface of NPs, providing steric stabilization and preventing aggregation. Poloxamer 188 and 407 are widely utilized in oral, parenteral, ocular, and topical applications. Poloxamer 188-modified annonaceous acetogenins NS were found to be stable in various physiological media and exhibited a sustained drug release [41]. It was shown that the natural hydrocolloid alginate would effectively stabilize NCs by offering both electrostatic and steric stabilization, requiring a significantly lower concentration compared to commonly used stabilizers either in a solid or liquid state [42]. An investigation was conducted to develop luteolin-loaded NS using the antisolvent precipitation/sonication process, examining the impact of four stabilizers: two nonionic stabilizers (Pluronic F127 and Tween 80) and two polymeric stabilizers (hydroxypropyl methylcellulose (HPMC) and alginate). Among these, alginate-stabilized NS exhibited the smallest particle size, highest ZP, and superior stability due to its dual stabilizing effects (electrostatic and steric). Conversely, Pluronic F127 improved luteolin’s pharmacodynamic effectiveness and skin delivery [40]. Stabilizers with a minimal impact on drug solubility are often the initial choice for NS preparation. A study demonstrated that stabilizers like polyvinylpyrrolidone (PVP) K30, Pluronic F68, and HPMC had a minimal impact on ibuprofen solubility, leading to stable NC suspensions. In contrast, sodium laureth sulfate, Tween 80, and Pluronic F127 increased the ibuprofen solubility, causing NS instability and particle size growth during storage [43]. TPGS, a water-soluble esterified tocopherol derivative, is a versatile stabilizer and solubilizer with high physical stability and low toxicity [44]. High physical stability, an amphiphilic nature, permeation glycoprotein inhibitory activities, and solubilization effects make TPGS preferable to use as a stabilizing agent in oral, ophthalmic, and parenteral formulations [45]. Furthermore, TPGS acts as an antioxidant by releasing α tocopherol when hydrolyzed by cytoplasmic esterases, which protects cell membranes from damage caused by free radicals [46].

**Figure 1:**
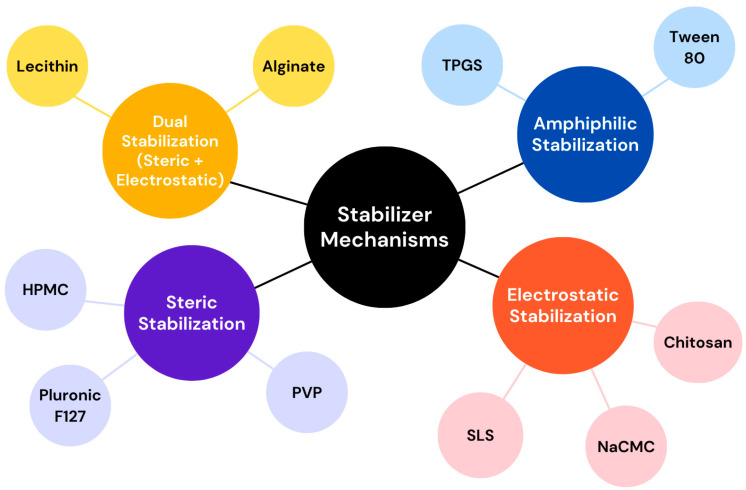
Flowchart illustrating different stabilization mechanisms of commonly used stabilizers. HPMC; hydroxypropyl methylcellulose, NaCMC; sodium carboxymethyl cellulose PVP; polyvinylpyrrolidone, SLS; sodium lauryl sulfate, TPGS; D-alpha-tocopheryl polyethylene glycol 1000 succinate (prepared with BioRender.com, accessed on 5 January 2025).

Amino-acid-based stabilizers, including leucine copolymers, arginine, proline, and transferrin, are effective in forming stable drug NCs [47]. Lecithin, a natural emulsifier is preferred for heat sterilizable parenteral formulations, while albumin serves as a stabilizer and targeting agent at varying concentrations [48]. In addition, natural compounds such as lentinan, β-glucan, rubusoside, and gypenosides have been identified as multifunctional stabilizers in the preparation of drug NS [49,50]. Cellulose derivatives like HPMC, hydroxypropyl cellulose, hydroxyethyl cellulose, and nanocrystalline cellulose provide steric stabilization through the surface adsorption of hydrophobic groups, enhancing the physical stability of NS [51]. Soluplus^®^, a polyvinyl caprolactam-polyvinyl acetate-PEG copolymer, is an innovative excipient that enhances the stability, dissolution rate, and bioavailability of NS [52]. Indomethacin NS, prepared using ultrasonic-assisted precipitation, was successfully stabilized with Soluplus^®^ [52]. Other hydrophilic polymers, including polyvinyl alcohol (PVA), PVP, as well as PEGylated chitosan, are also effective stabilizers [53]. Nonionic surfactants and polymers stabilize NS through steric hindrance, while ionic surfactants and polymers, like sodium carboxymethyl cellulose and chitosan, stabilize them via electrostatic repulsion. Drug NCs dispersed in water are unstable due to their high surface free energy, leading to aggregation, solidification, or crystal growth. Hydrophilic polymers effectively hydrate and stabilize NC surfaces by reducing the surface energy through strong interactions with water molecules [54]. Compatibility between the surface energies of drugs and stabilizers, assessed using static contact angle measurements, ensures stable and uniformly sized particles [55]. The classical Stokes Einstein equation indicates that nanosystem stability decreases with the rising temperature and increases with higher medium viscosity. However, high viscosity can hinder particle size reduction during NC preparation. Protein-based surfactants like hydrophobin enable surface functionalization for targeted delivery, leveraging genetic engineering for versatility in drug applications [56]. Surfactants and co-surfactants are essential in microemulsion-based NS preparation, influence phase behavior, drug solubility, and loading. Common surfactants include Tweens such as Tween 80 and sodium dodecyl sulfate (SDS), while co-surfactants like bile salts, transcutol, and alcohols enhance steric stabilization [57]. Due to safety profile regulatory acceptance, class III organic solvents, such as ethyl alcohol, isopropyl alcohol, ethyl acetate, and acetone, are preferred in emulsion solvent evaporation techniques [58]. Additionally, complexing agents like cyclodextrins improve drug dissolution and bioavailability. NS may also include buffers, preservatives, osmotic agents, and cryoprotectants, tailored to the drug’s properties and route of administration, ensuring an optimal performance and stability. Stabilizers with balanced hydrophilic and hydrophobic properties are essential for NC stability. Hydrophobicity aids surface adsorption for dispersion, while hydrophilicity prevents aggregation by interacting with water. Charged hydrophilic molecules further enhance stability through electrostatic repulsion. An in-depth study emphasizes the importance of amphiphilic excipients with long hydrophobic alkyl or polymer block chains for stabilizing drug NS [59]. After screening 28 excipients, it was found that traditional parameters, like the molecular weight and HLB value, are insufficient predictors of excipient effectiveness. Instead, the length and conformational flexibility of the hydrophobic regions in excipients were identified as critical factors for stability. This conclusion was supported by molecular modeling studies on excipient drug NP interactions. Selecting an effective stabilizer for long-term stability in pharmaceutical NS remains challenging. Computational and molecular dynamic simulations, coupled with topology and powder XRD analyses, provide valuable insights into crystal lattice formation and fracture mechanisms, aiding in understanding stabilization processes. For instance, these methods have elucidated the NC formation of glimepiride and the nanocomposite formation of agomelatine via wet media milling [60,61]. A flowchart illustrating the different stabilization mechanisms of commonly used stabilizers is presented in Figure 1.

## 4. Preparation Methods

Pharmaceutical NS are prepared using various manufacturing methods, each with its own significance and applications. NPs are commonly made by compressing or aggregating particles from small molecules to nanosized particles (bottom-up approach), or by reducing or dispersing big particles to the nanosized range (scale-down technology).

### 4.1. Scale-Down Technologies

#### 4.1.1. High-Pressure Homogenization (HPH)

**Figure 2:**
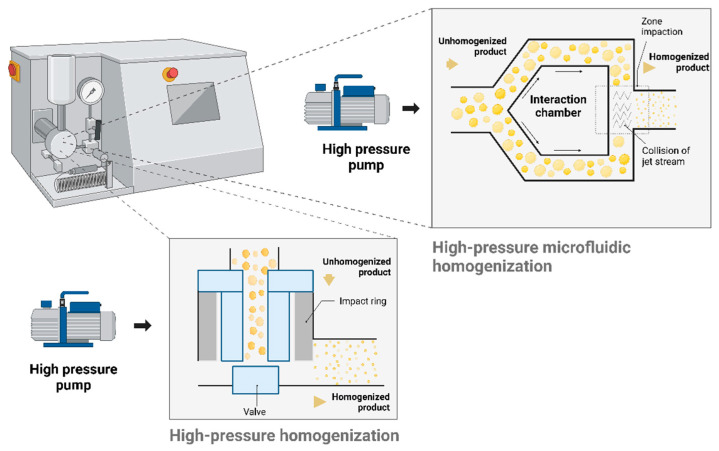
Schematic diagram illustrating high-pressure homogenization and microfluidization process (adapted from [71], published by MDPI, 2024).

In the homogenization process, particle size reduction is achieved by passing a pre-milled suspension of microsized drug particles suspended in a particular surfactant solution through a valve with a narrow aperture under high pressure (100–1500 bars) [62]. This rapid fluid velocity change increases the dynamic pressure while decreasing the static pressure, resulting in cavitation. The implosion forces and shock waves generated during cavitation, along with shear forces from particle collisions and the impact of high-speed fracture, reduce the microparticles (≤25 μm) to the nanoscale. Inherent crystal defects in the particles further facilitate their breakdown [63]. This method is employed in devices like the APV Gaulin Micron LAB 40 homogenizer (APV Homogenizer, Lübeck, Germany) and the NS 100 1L-Panda 2K high-pressure homogenizer (NIROSUAVI S.P.A., Parma, Italy). The two key homogenization techniques used are microfluidization and piston gap homogenization (Figure 2). Microfluidization relies on a jet stream mechanism, where the coarse suspension is directed through homogenizing chambers of either a “Z” or “Y” design. In piston gap homogenization, the suspension passes through a fine gap at a very high velocity. These processes typically operate at pressures ranging from 500 bars to 350 MPa [64,65]. This process employs high-speed collision, shear, and cavitation forces to achieve particle size reduction. In optimizing HPH, three primary process variables are crucial: the number of pass cycles, the applied pressure, and the process temperature. On the formulation side, key factors include the type and concentration of the surfactant used [66]. HPH is a well-established, scalable, and cost-effective method for producing pharmaceutical NS, offering significant advantages in particle size control and nonthermal processing. However, scaling up at the industrial level presents several challenges, such as high energy consumption and the need for the careful optimization of various processing parameters. While the method is highly applicable to certain drug classes, its use for highly viscous or complex formulations may be limited, highlighting the importance of carefully evaluating its suitability for each specific application. Moreover, operating machinery at high pressures or over numerous cycles for extended periods can result in the wear and tear of components. Research indicates an inverse relationship between the homogenization pressure and the number of cycles with particle size [67,68]. Achieving the desired mean particle size and uniformity often requires multiple passes through the homogenizer, depending on the drug’s hardness and the target properties. High-pressure homogenizers are available in various capacities, ranging from 40 mL for laboratory use to several thousand liters for large-scale manufacturing. Pre-milling with high-speed homogenization prevents the blockage of the homogenization gap in the microfluidic chamber by decreasing the initial particle size, ensuring a smoother flow and minimizing the risk of clogging during the subsequent HPH process. An evaluation of the high-speed homogenization and HPH processes was undertaken, focusing on critical process parameters such as the homogenization speed, time, pressure, and cycles to assess their impact on the critical quality attributes (CQAs), particle size, polydispersity index (PDI), and ZP of flurbiprofen NS stabilized with surfactant, Plantacare 2000^®^ [69]. The optimized conditions: 10,000 rpm for 10 min (high-speed homogenization) and 30,000 psi (HPH) successfully produced the drug NS. This study concludes that a single high-pressure application is sufficient to achieve the smallest particle size for flurbiprofen NS, offering a time-saving advantage compared to sequential pressure or pre-milling applications. The drug NS demonstrated a particle size, PDI, and ZP of 738.97 ± 14.93 nm, 0.14 ± 0.03, and −36.67 ± 0.38 mV, respectively. This finding aligns with previous studies, which suggest that increasing the number of homogenization cycles at a high pressure reduces the particle size [70]. In addition, the NS maintained physical stability for 12 months when stored at 25 ± 2 °C and for 6 months at 40 ± 2 °C.

Viscosity enhancers play a significant role in the nanosizing process by increasing the particle density in the dispersion region, preventing crystal growth, and enhancing the overall efficiency of homogenization [72]. During the process, metastable amorphous particles produced by precipitation methods can be transformed into stable crystalline forms. Achieving the desired particle size typically requires multiple homogenization cycles. This method offers several advantages, including ease of scaling up, compatibility with both dilute and concentrated suspensions, minimal contamination risk, and suitability for aseptic manufacturing processes. However, it also has certain limitations, such as the prerequisite for micronized particles, the need for repeated cycles, high energy demands, and the risk of contamination from the metal components of the equipment. Many homogenization technologies are either patented or have patents pending. Examples include Hydrosol (Novartis, Patent No. GB 2269536), Nanocrystal™ (Elan Nanosystems, Patent No. US 5145684), Dissocubes^®^ (Skye Pharma, Patent No. US 5858410), Nanopure (Pharma Sol, Patent Application No. PCT/EP00/0635), and NANOEDGE™ (Baxter, Patent No. US 6884436). Despite the potential of nanosized co-crystals, research combining nanotechnology and co-crystals is still in its early stages. A study compared HPH and high-power ultrasound to formulate nanosized co-crystals of 4-aminosalicylic acid and sulfamethazine, evaluating differences in the size, morphology, polymorphic form, and the dissolution enhancement of the formed co-crystals [7]. Both methods developed highly crystalline-form I co-crystals. HPH resulted in smaller, nanosized, needle-like co-crystals with a narrow size range, especially at higher pressures, while ultrasound yielded microsized co-crystals with a different morphology. Co-crystals prepared by HPH showed the greatest improvement in dissolution, though both nano- and micro-sized co-crystals enhanced sulfamethazine’s dissolution.

HPH presents a well-established, scalable, and cost-effective method for producing pharmaceutical NS, with significant advantages in terms of particle size control and nonthermal processing. However, it faces challenges such as high energy consumption, equipment wear, and the need for careful optimization, particularly for large-scale applications. While the method is highly applicable for certain drug classes, its use for highly viscous or complex formulations may be limited, making it important to carefully evaluate the suitability of HPH for each specific application.

#### 4.1.2. Media Milling Method

**Figure 3:**
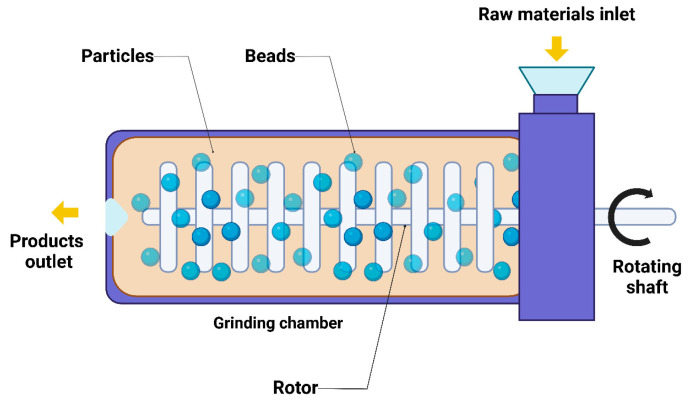
Schematic diagram illustrating media milling process (prepared with BioRender.com, accessed on 25 November 2024).

This technique, also identified as wet stirred media milling (WSMM), nanosizing, nanomilling, nanonization, or wet bead milling, is considered the most favored method for producing drug NS [7,73]. According to this method, NS are formulated using a high-shear colloid mill, ball mill, or media mill. A colloid mill works on the principle of shearing, where the material is introduced into the mill and processed through a rotor stator mechanism. The rotor spins at high speeds, creating intense shear forces as the material passes through the narrow gap between the rotor and stator (Figure 3). This results in the particle size reduction, homogenization, and dispersion of the material, producing fine suspensions. The colloid mill offers several benefits, such as ease of cleaning, high efficiency with minimal requirements, no need for pressure to achieve size reduction, and easy adjustability, although it may experience wear on the rotating plates. In a ball mill or in wet media milling, size reduction occurs through impact and attrition within a chamber containing the drug and stabilizer as well as water. This method is appealing due to its low energy consumption, simplicity of scaling up, minimal batch-to-batch deviation, and capacity to process bulk amounts, as demonstrated by four FDA-approved drugs. The quantity of the material in the mill is critical; an excessive feed leads to a cushioning effect, whereas an insufficient amount lowers productivity and heightens the abrasive wear on the mill components [74]. Using strongly crosslinked polystyrene resin milling media has solved this problem. However, prolonged milling times may increase the amorphous fraction in the product, potentially causing instability. Additionally, a wet milling process utilizing small, hard zirconium dioxide beads to produce uniformly sized NS has been described [75]. An X-ray diffraction (XRD) examination indicated that the crystalline nature and structure of phenytoin continued unchanged in spite of the wet milling. Furthermore, advancements in milling techniques, such as high-energy ball milling, have shown the potential to enhance the mechanical properties of the milled materials. The input of surfactants and stabilizers during the milling process can also help to maintain stability and prevent NP agglomeration.

The enhanced dissolution and oral bioavailability of a BCS II drug, probucol, in a NS form, have been achieved using the wet milling technique [76]. Planetary bead milling equipment was employed to produce nanosized probucol NPs/NCs in an aqueous medium, utilizing a ternary stabilizer system consisting of a primary stabilizer (hydroxypropyl cellulose, HPMC, methyl cellulose), a nonionic surfactant (Pluronic^®^ F68), and an anionic surfactant (SDS). The probucol NS exhibited good physical stability after storage for over 7 days at both 4 °C and 25 °C. Among cryoprotectants, the solidified probucol NS with trehalose demonstrated the highest dissolution rate (>60% within 2 h) in comparison to other cryoprotectants. Pharmacokinetic studies revealed that the probucol NS had an approximately 15-fold higher AUC value compared to the coarse probucol suspension following oral administration to rats.

Despite several advantages, WSMM faces processing and operational challenges, including an energy-intensive operation, high costs, extended operating time, and contamination of actives by the beads [77]. Additionally, this technique may not be suitable for all types of drugs, particularly those with high viscosities or complex formulations that require careful stabilization. For certain drugs, a combination of media milling with other techniques, such as stabilizer optimization or post-milling processing, may be required to enhance the product stability and performance. Despite these challenges, WSMM is a valuable technique for the industrial-scale production of NS when appropriately optimized. Various researchers have employed different modeling approaches such as empirical regression and a response surface methodology to study the WSMM process. Recent research investigated the effects of the stirrer speed and bead material loading on fenofibrate particle breakage during WSMM using three kinetic models and a microhydrodynamic model [73]. The progression of the particle size was monitored using laser diffraction while WSMM was conducted at speeds of 3000–4000 rpm with polystyrene or zirconia beads at concentrations of 35–50% (*v*/*v*). This study found that the nth-order model, particularly near second-order kinetics (n ≅ 2), best described the temporal evolution of particle breakage. Faster breakage occurred at greater stirrer speeds and/or with the increased loading of zirconia beads compared to polystyrene beads. A statistically significant multiple linear regression model (*p* ≤ 0.01) of three microhydrodynamic parameters most effectively elucidated the changes in the breakage rate constant, with an R^2^ value of 0.99 or higher. The nth-order kinetic–microhydrodynamic correlation provides a strong predictive capability, surpassing the accuracy of purely empirical correlations.

#### 4.1.3. Dry Co-Grinding

**Figure 4:**
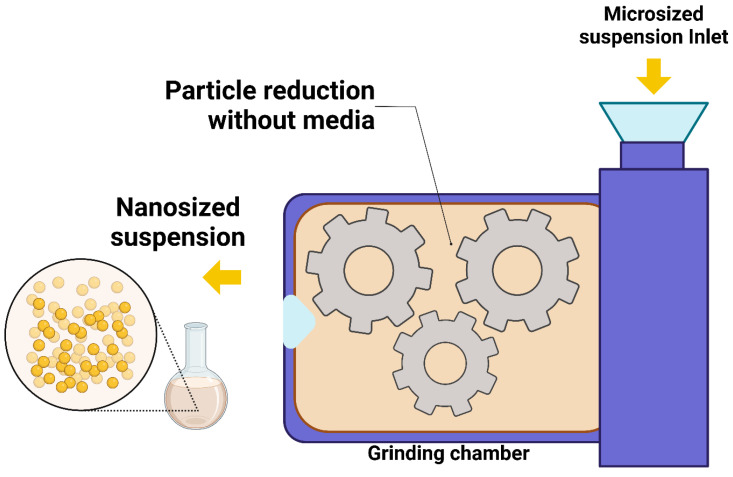
Schematic diagram illustrating dry co-grinding process (prepared with BioRender.com, accessed on 25 November 2024).

The preparation of stable NS using the dry co-grinding technique has been explored with various polymers and copolymers, including PVP, HPMC, PEG, SDS, and cyclodextrin derivatives [76]. In contrast to wet grinding methods, dry grinding is more cost effective, avoids toxic solvents, and simplifies the overall process while improving the drug’s performance and stability. However, the method has some limitations, including the potential for agglomerate formation and drug degradation due to mechanical stresses. Controlling the particle size and distribution requires the careful optimization of parameters, and the method may not be effective for highly viscous or difficult-to-grind drugs. This technique notably enhances the surface polarity and transforms a substantial portion of the drug’s crystalline state into an amorphous form [78]. The schematic representation of the dry co-grinding technique is presented in Figure 4.

The successful preparation of stable NS has been reported by dry grinding drugs which have low solubility with soluble polymers and copolymers, followed by dispersion in a liquid medium. The co-grinding process of crystalline meloxicam with amorphous PVP or semi-crystalline PEG, optimized using a three-level full factorial response surface design, was reported [79]. The ideal conditions for PVP were a 1:1 meloxicam to PVP-C30 ratio with a rotation frequency of 400 rpm. In the case of PEG 6000, the ideal size reduction was accomplished at 1:2 ratio and 400 rpm, resulting in NPs averaging 174 nm in diameter. XRD revealed that the optimized PVP-C30 compositions contained amorphous meloxicam NPs, while PEG 6000 samples also had meloxicam NCs. The dissolution properties of the amorphous product were considerably enhanced under nasal conditions (pH 5.1, 30 °C), suggesting that these dry powder formulations could be promising for systemic nasal drug delivery. A study compared the effects of dry and wet grinding on the yield, physico-chemical properties, and solubility of nimodipine NPs [80]. Using the nimodipine-to-HPMC ratio of 1:0.6, the NPs were prepared via both methods and subjected to various in vitro characterizations. The solubility of pure nimodipine was 0.339 µg/mL, which increased to 1.948 µg/mL with the physical mixture, 3.367 µg/mL with dry grinding, and 19.952 µg/mL with wet grinding. After 60 min, the dissolution rates were 33.95% for pure nimodipine, 39.48% for the physical mixture, 49.80% for dry grinding, and 56.48% for wet grinding. This study concluded that nimodipine HPMC NPs, especially those produced by wet grinding, significantly improved nimodipine’s solubility and dissolution rate.

Microneedles are tiny, minimally invasive needles designed to pierce the outer layer of the biological membrane, enabling drug delivery without causing significant pain or discomfort. Dissolving microneedles (DMNs) is a subtype of microneedles made from biocompatible, water-soluble polymers. DMNs eliminate the need for removal, offering a safer and more convenient method for drug delivery. DMNs combined with NS and co-grinding were used to address the low solubility of ketoprofen [81]. Ketoprofen NS were developed using PVA at concentrations of 0.5%, 1%, and 2% while co-grinding involved grinding ketoprofen with PVA or PVP at various drug-to-polymer ratios. The most effective microneedle formulations, F5-MN-NS and F11-MN-CG, achieved cumulative drug permeation of 3.88 ± 0.46 µg and 8.73 ± 1.40 µg, respectively, in 24 h. Thus, combining DMNs with NS or co-grinding systems seems to be an encouraging method for the transdermal delivery of ketoprofen.

#### 4.1.4. Emulsion Solvent Evaporation

**Figure 5:**
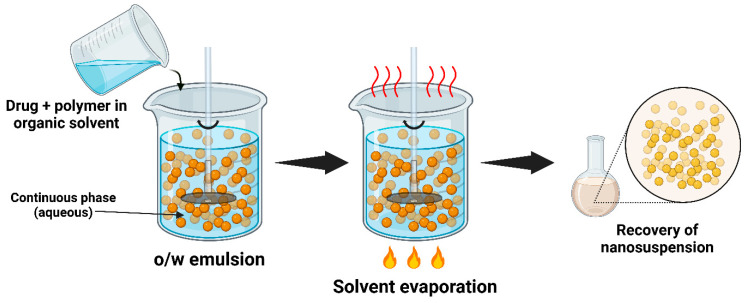
Schematic diagram illustrating emulsion solvent evaporation process (prepared with BioRender.com, accessed on 25 November 2024).

In the emulsion solvent evaporation technique, the process begins with dissolving the drug in a volatile, water-immiscible organic solvent like dichloromethane or chloroform. This drug solution is then emulsified into an aqueous phase containing a stabilizer or surfactant, such as PVA or lecithin, to create an oil-in-water emulsion. The emulsification is typically achieved through high-speed homogenization or ultrasonication, ensuring the uniform distribution of the drug within the emulsion droplets [82]. Following this, the emulsion undergoes solvent evaporation under continuous stirring, often under a reduced pressure or elevated temperatures, which results in the formation of drug NPs as the solvent evaporates (Figure 5). These NPs are stabilized by the surfactant. The resulting NS is then collected and, if necessary, further processed into a dry powder form through techniques like lyophilization or spray-drying. The emulsion solvent evaporation technique offers advantages such as precise control over the particle size, scalability for industrial production, and versatility for different drug types. However, challenges include the need for the careful handling of organic solvents and ensuring the long-term stability of the NS. Important factors to consider during this process include the globule size and the concentration of the stabilizer. Despite these challenges, this technique remains a valuable tool in preparing formulations of drugs with low aqueous solubility [83].

Ursolic acid NPs were formulated by the emulsion solvent evaporation technique, followed by freeze-drying, to improve their water solubility as well as oral bioavailability [84]. During the process, the drug-loaded nanoemulsion had an average particle size of 69.7 nm with a PDI of 0.005. After solvent removal via rotary evaporation, the resulting NS had a size of 100.2 nm with the same PDI. The freeze-dried NPs showed a size of 157.5 nm, PDI of 0.005, and ZP of 20.33 mV. Characterization through various techniques established the amorphous form of ursolic acid in the NPs. Solubility tests demonstrated that these NPs significantly improved the equilibrium solubility in gastric (13.48-fold) and intestinal (11.79-fold) fluids than the pure drug. Additionally, the dissolution rates in both simulated fluids were enhanced, leading to a 2.68-fold enhancement in the bioavailability. The antioxidant activity of the prepared NPs also improved, with an EC_50_ reduction of 37.5 times compared to the raw drug. In another study, a 1% voriconazole-loaded NS was formulated through a quasi-emulsion solvent evaporation process using Eudragit RS 100, with Pharmasolve^®^ as a corneal permeation enhancer [85]. Characterization using Zetasizer and microscopy revealed uniform, spherical NPs which were nano-sized (~138 nm), had good entrapment efficiency (~98.6%), and suitable ZP (22.5–31.2 mV), signifying greater stability. Both in vitro and in vivo tests showed that the NS enhanced permeability compared to a commercial voriconazole injection.

The emulsion solvent evaporation method is scalable, but its efficiency decreases when transitioning from the laboratory to industrial scale due to challenges in solvent removal and controlling particle size distribution. Unfortunately, its industrial applicability may be limited by the use of organic solvents and the need for precise emulsion stability control; however, it remains a viable option for certain drug classes, especially when other methods like HPH or WSMM are less effective.

#### 4.1.5. Sonocrystallization

**Figure 6:**
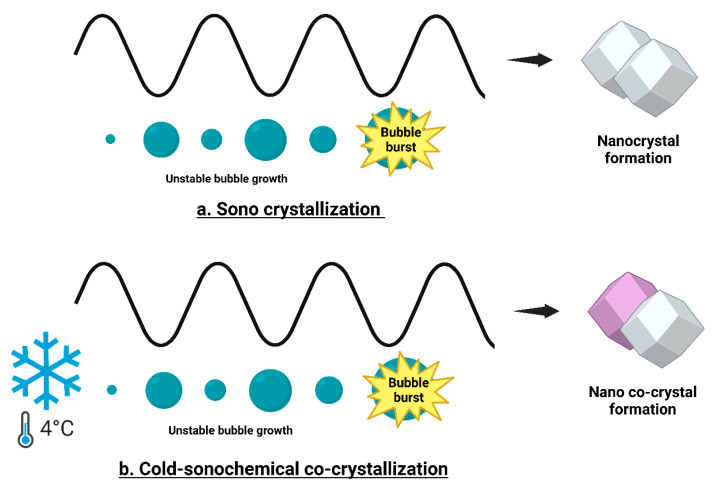
Schematic diagram illustrating the sonocrystallization process (prepared with BioRender.com, accessed on 25 November 2024).

Sonocrystallization is a unique technique utilized for the formulation of stable NS and nanoemulsions. This method employs ultrasound, typically within a frequency range of 20 to several MHz, to induce and control the crystallization process of pharmaceutical actives, resulting in the formation of nanosized particles. This technique has gained popularity as a result of enhanced particle size reduction and ensuring uniform size distribution, which are critical factors in drug delivery systems. In this technique, ultrasonic waves create cavitation within the solution, leading to improved micromixing, higher mass transfer rates, and consistent supersaturation (Figure 6). Ultrasound-induced cavitation aids in size reduction by releasing energy from the implosion of cavity bubbles, which generates high temperatures and pressures that enhance nucleation [86]. Moreover, as ultrasonic waves propagate through the medium, they create streaming and shock waves, leading to particle breakage in nearby areas and the formation of a greater number of nuclei.

This process shortens the induction time, decreases the width of the metastable zone, boosts nucleation rates, results in smaller and more uniformly sized particles, and also avoids seeding crystals [87]. This intense environment promotes the nucleation process and breaks down larger crystals into finer particles, leading to the formation of nanoscale emulsions or suspensions. The process is particularly effective in controlling the nucleation and crystallization processes, ensuring the production of stable and uniform particles. To enhance the stability and control the particle size and distribution, it is crucial to regulate the nucleation rate and prevent particle growth or agglomeration through steric or electrostatic stabilization. Rapid nucleation is possible to achieve by inducing a high proportion of supersaturation in a short duration, allowing the process to quickly surpass the metastable zone, thereby prioritizing nucleation over crystal growth [88]. This technique allows the production of NPs with significantly reduced sizes, which improves the solubility and bioavailability of low-aqueous-soluble drugs. This technique was employed to enhance the solubility and dissolution rate of the poorly water-soluble BCS Class II aldosterone receptor antagonist, eplerenone [89]. The optimized formulation achieved a saturated solubility of 1.29 mg/mL in distilled water and slightly higher in pH 1.2 media (1.86 mg/mL). The size of the drug particles was reduced to 133 nm with a PDI of 0.824, showing a minimal discrepancy between the observed as well as predicted values based on a three-factor, two-level multifactorial design. The formulation released 91% of the drug within 10 min and achieved complete release within 45 min, significantly outperforming the raw drug in the release rate.

However, this method requires significant energy input, which may not be cost-effective for large-scale production. The process can generate heat due to the intense cavitation, which may degrade thermolabile drugs or require additional cooling systems. The need for specialized ultrasound equipment can increase the initial setup costs for pharmaceutical manufacturers. Recent research has focused on optimizing the sonocrystallization parameters, such as the frequency, amplitude, and duration, to enhance the efficiency and scalability of the process. Advances in the equipment design have also led to more energy efficient and controllable ultrasound devices, making the technique more viable for large-scale production. Recent studies have explored the integration of sonocrystallization with other nanoformulation techniques, such as microfluidization and HPH, to further improve the quality and stability of nanoemulsions [90]. These studies highlight the growing interest in sonocrystallization as an innovative technique in the formulation of nanoemulsions for drug delivery applications. By understanding and leveraging the benefits of this method, researchers and pharmaceutical companies can develop more effective and stable NS. The recrystallization technique was evaluated for the particle size reduction of azithromycin using both cooling and antisolvent crystallization methods with ultrasonic irradiation. Ultrasound-assisted antisolvent crystallization at temperatures below 10 °C resulted in an 80% reduction in particle size, with a narrower distribution and higher yield, all achieved within 5 min of sonication. Optical and electron microscopy images revealed a uniform rod/plate-shaped geometry and XRD analysis confirmed an increase in the amorphous nature of the drug.

Cold sonochemical co-crystallization has been effectively used to produce nano-co-crystals [91]. Traditional one-solvent and two-solvent approaches include dissolving all ingredients either in one solvent or in two separate solvents before adding the drug solution in the antisolvent while applying an external energy source like ultrasonic waves (Figure 6). A novel pseudo one-solvent bottom-up cold sonochemical synthesis approach, employing equimolar concentrations of surfactants (SDS and TPGS), was used to prepare stable nano-co-crystals of lamivudine and zidovudine [92]. In vitro characterization showed a particle size under 1000 nm, PDI below 0.500, and ZP under −30 mV. Moreover, cytotoxicity studies demonstrated that the developed nano-co-crystals diminished the toxicity of both drugs to HeLa cells.

Sonocrystallization is still primarily a research scale technique, with limited industrial implementation. While it holds promise for specific applications, such as the preparation of heat-sensitive drugs or when particle size control is paramount, its scalability and throughput limitations hinder its widespread industrial adoption. The method may be most applicable for niche drug products or in combination with other techniques to optimize particle size and improve efficacy.

#### 4.1.6. Supercritical Fluid Technique

The SCF technique is an advanced method employed in the preparation of NS, which leverages the unique properties to produce particles with enhanced solubility, particularly for poorly water-soluble drugs. The SCF technique can be implemented in various ways, including a supercritical antisolvent, the rapid expansion of supercritical solutions, SCF extraction of emulsion, and the gas antisolvent method. SCF techniques for preparing NS offer distinct advantages and limitations. The supercritical antisolvent method allows control over the particle size and is suitable for thermolabile compounds, but is limited by the solubility of compounds in supercritical CO_2_. The explosive growth of supercritical solutions produces small, uniform particles with minimal residual solvents, though it is restricted to compounds highly soluble in CO_2_ and may lead to particle agglomeration. The SCF extraction of emulsion is effective for forming nanosized particles with high drug loading, but it involves complex and costly multistep processes. To enhance the mass transfer of organic solvents, the SCF extraction of an emulsion in a microfluidic system utilizing supercritical CO_2_ has been developed [93]. Lastly, the gas antisolvent method is suitable for large-scale production and generates high-surface-area particles, but can result in non-uniform particles and is not compatible with all solutes and solvents.

**Figure 7:**
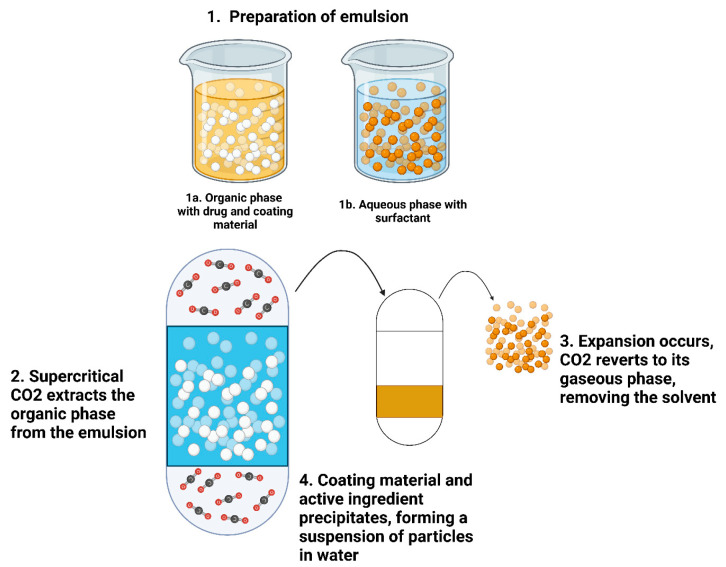
Schematic diagram illustrating supercritical fluid process (prepared with BioRender.com, accessed on 25 November 2024).

The SCF technique offers several advantages in NS preparation such as precise control over the particle size and distribution, minimal to no solvent residues, relatively low temperatures which are used here and are suitable for handling thermolabile compounds, and ease of industrial scale-up [94]. Supercritical CO_2_ was employed as a washing and drying medium for surface-modified NPs. A schematic representation of NS preparation using supercritical CO_2_ is presented in Figure 7. Supercritical CO_2_ technology has been recently applied to NS, including decanoic acid-modified Fe_3_O_4_ NPs [95]. Remarkably, this process achieved a decanoic acid removal efficiency exceeding 99%, indicating near-complete purification. This may arise from the high diffusivity of supercritical CO_2_. Despite its advantages, the SCF technique has certain limitations: the need for high-pressure equipment and the energy required to maintain supercritical conditions, the limited solubility of many drugs in supercritical CO_2_, and complex machinery requiring specialized expertise for its operation and maintenance. Recent advancements in SCF techniques have focused on improving the efficiency, scalability, and applicability by combining SCF with other NP production methods (such as milling or sonication) to enhance particle size reduction and distribution.

The NS of poorly water-soluble antioxidants such as genistein, quercetin, and caffeic acid, extracted from *Asplenium scolopendrium* leaves, was prepared using the sudden enlargement of a supercritical solution into the aqueous solutions method [96]. The statistical experimental approach utilizing a central composite design was used to investigate the influence of variables such as the oven temperature, pressure, CO_2_ flow rate, and modifier volume on the antioxidant activity index during this process. The results showed that this approach reduced particle agglomeration and improved the antioxidant capacity, indicating that this method could significantly enhance the bioavailability of herbal medicinal products in biological systems.

The SCF technique offers several advantages, including minimal solvent use, precise control over the particle size, enhanced drug solubility, and the preservation of heat-sensitive compounds. However, its scalability and industrial applicability are limited due to high equipment and operational costs, challenges in process control, and low throughput. While the technique is ideal for small-scale or specialized drug formulations, it faces challenges in large-scale manufacturing, making it more suitable for niche applications rather than mass production. Despite these limitations, SCF remains a promising method for advanced drug delivery systems where particle size control and environmental concerns are prioritized.

#### 4.1.7. Pulsed Laser Ablation in Liquid (PLAL)

Particle engineering techniques, like pulsed laser ablation and PLAL, offer innovative methods for producing micro- and NPs with particle sizes ranging from 10 µm to 10 nm [97,98]. It is a rapid, clean, and accurate dry or wet grinding technique that employs highly energized, focused laser pulses to irradiate drug pellets either alone or in ultrapure water in a closed chamber. This process triggers microexplosions at the surface, causing ablation and the formation of nanoscale drug particles. Laser wavelengths of 248 nm, 1064 nm, and 532 nm, covering the UV-VIS-IR spectrum, were used for reducing the particle size of ibuprofen, meloxicam, and niflumic acid via the pulsed laser ablation technique, with the ablated particles carried by a nitrogen gas jet stream and subsequently filtered [99]. The laser fluence, ranging from 1.5 to 12 J/cm^2^, was maintained just above the ablation threshold to ensure effective ablation. The particle size distribution of ablated ibuprofen, niflumic acid, and meloxicam were below 100 nm, 153 nm, and 240 nm, respectively. Laser ablation techniques provide advantages such as high precision, rapid deposition, particle purity, uniform size distribution with minimum degradation, and physicochemical changes [100,101]. In the past decade, compounds like vanadyl phthalocyanine, quinacridone, and beclomethasone dipropionate have been successfully formulated into NS using this method.

The production of meloxicam particles using the PLAL technique has been described [101]. In this method, crystalline meloxicam powder was compressed into targets and irradiated with a nanosecond neodymium laser in distilled water. The process produced particles ranging from approximately 100 nm to 10 µm, with an average size of 1.0–2.0 µm, which is significantly smaller than the original material. The analysis confirmed that the chemical composition of meloxicam remained intact, though partial amorphization occurred. The fragmentation of the pressed target due to laser-induced recoil forces was identified as the primary mechanism for particle formation. Another researcher utilized PLAL to examine the impact of different laser wavelengths, energy densities, and polymers (PVP, PVA, and Poloxamer) on the drug’s particle shape, structure, solubility, and in vitro properties [100]. The process generated nearly spherical amorphous particles between 60 and 700 nm. PLAL significantly enhanced meloxicam’s solubility (from 0.0203 mg/mL to 0.0797 mg/mL), boosted its dissolution rate (~85%) and produced an intermediate product suitable for various drug delivery methods, including pulmonary, nasal, and transdermal administration. PLAL was applied to fenofibrate and naproxen, revealing that the use of a 1064 nm wavelength laser produced drug particles with a size distribution between 0.5 and 5 μm [102].

While PLAL is a promising method for NS preparation due to its precision, ability to handle heat-sensitive drugs, and solvent-free nature, its scalability and industrial applicability is hindered by high costs, energy consumption, and complexity. Thus, PLAL is more suited for research and small-scale applications, with ongoing developments needed to overcome its limitations for large-scale commercial use.

### 4.2. Scale-Up Technologies

#### Precipitation Technique

**Figure 8:**
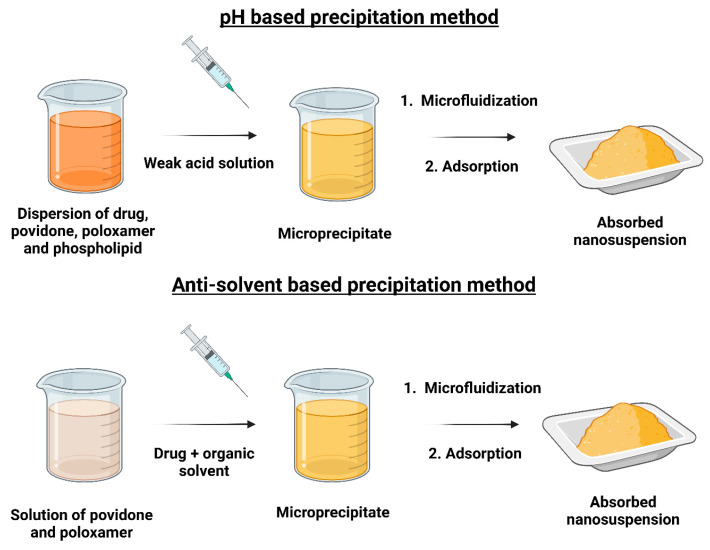
Schematic diagram illustrating precipitation technique (prepared with BioRender.com, accessed on 25 November 2024).

Precipitation techniques involve aggregating materials of a sub-colloidal dimension into colloidal-sized particles by pH-based or antisolvent nanoprecipitation methods. Precipitation is achieved by creating a supersaturated drug solution in a water-miscible organic solvent at an optimal temperature and then dispersing it in water, a nonsolvent, under rapid stirring [103]. The pH-based nanoprecipitation method is a versatile approach for preparing NPs by exploiting changes in solubility induced by pH adjustments [104]. In this method, a pH-sensitive polymer or drug is dissolved in a solvent at a specific pH where it remains soluble, and upon rapid mixing with an aqueous phase of contrasting pH, its solubility decreases, leading to the formation of nanosized particles (Figure 8). It is a simple, solvent-free technique suitable for lipophilic and lipophobic drugs.

The principle behind the antisolvent precipitation method is based on the controlled precipitation of a solute when a solvent, in which the solute is dissolved, is rapidly mixed with a nonsolvent (Figure 8). This process depends on the solubility difference of the solute in two solvents, where the change in solvent induces high supersaturation, prompting rapid nucleation while preventing supersaturation near the forming crystals. According to the classical Ostwald law of nucleation, rapid nucleation coupled with a slow growth rate is essential for forming a thermodynamically stable crystal form [105]. High supersaturation conditions often lead to the formation of acicular or needle-like crystal habits. These elongated crystals are more prone to fragmentation, which promotes the formation of additional nuclei at the expense of further crystal growth. Additionally, the absence of impurities during the process minimizes lattice imperfections or defects, resulting in more uniform crystal structures. These defects, when present, can be exploited during homogenization to break down the particles to the nanometer scale, enhancing the efficiency of the particle size reduction process. The key factors that influence this process are the rate of mixing, the concentration of the solute, and the properties of the solvents used. To stabilize the NPs and prevent agglomeration, surfactants or stabilizers are introduced, and techniques like sonication or HPH may be applied to ensure a uniform particle size distribution. The right amount of surfactant or selective crystallization inhibitors must be used to inhibit crystal formation. The suspension is then filtered to remove any larger particles or impurities, and further purification steps may be employed to eliminate residual solvents. This technology was utilized to produce amorphous drug NPs by the pharmaceutical industry to develop Nanomorph™ [106].

A study reported developing glimepiride NS using different methods to enhance their solubility [107]. Twelve preparations were created using a combination method involving antisolvent precipitation followed by sonication, while six products were prepared by the nanoprecipitation method. The combination method showed a superior performance with a higher entrapment efficiency (82.04%), smaller particle size (129–180 nm), good ZP (30.16 mV), and better drug release (86.76%) compared to the nanoprecipitation method, making it the preferred technique for preparing drug NS.

The precipitation technique for preparing pharmaceutical NS is simple, cost-effective, and scalable, making it suitable for large-scale industrial production. It can be applied to a wide range of drugs, including poorly water-soluble compounds. However, it faces challenges such as difficulty in controlling the particle size and distribution, risk of agglomeration, and potential use of organic solvents. The need for precise control and additional stabilizers can complicate the process. Despite these limitations, the method remains a promising option for mass production, particularly when solvent use is minimized and environmental considerations are taken into account. Different NS preparation techniques, including their advantages, limitations, energy consumption, scalability, and particle size uniformity, are summarized in Table 1.

## 5. Administration Routes

### 5.1. Oral Nanosuspension

Oral NS are designed to improve the solubility, dissolution rate, and bioavailability of poorly water-soluble drugs in BCS Class II and IV. By decreasing the particle size to the nanometer scale, NS significantly increase the surface area of the drug, leading to an increase in saturation solubility and faster dissolution. This causes a higher concentration gradient in the gastrointestinal tract, resulting in enhanced drug absorption and bioavailability. Furthermore, NS provide versatility in drug formulation and help to overcome the food effects that typically affect oral drug absorption. An investigation was undertaken to assess the in vivo kinetics of ritonavir NS in rats (fed and fasted conditions) and was compared to various formulations [108]. Under fed conditions, the NS with an average particle size of 545 nm increased C_max_ by 8.9 times and AUC_0−t_ by 12.5 times that of the coarse powder, and by 1.9 and 2.1 times that of the commercial product (Norvir^®^), respectively.

Oral suspensions are highly preferred for geriatric and pediatric populations due to their liquid form, which ensures chemical stability and ease of administration. A novel NS of benznidazole, a class II drug aimed at the pediatric population for Chagas disease, was produced using an organic solvent-free nanomilling approach [109]. The prepared NS exhibited a particle size of <500 nm, an acceptable PDI (0.23), high ZP, and physical stability for at least 90 days. The formulation showed tenfold higher solubility and exhibited improved in vitro dissolution behavior, toxicity profiles, and efficacy against *Trypanosoma cruzi* than pure drugs. NS also offer the benefit of masking the taste of drugs and prolonging the drug action. A successful preparation of a taste-masked phospholipid stabilized tacrolimus NS using the microfluidization method has been reported [110]. A spectral analysis revealed interactions between tacrolimus and phospholipids, while a thermal analysis showed tacrolimus transformed into an amorphous state within the nanodispersion. The dissolution rate was significantly improved (35-fold after 0.5 h and 15-fold after 2 h). The nanodispersion significantly reduced gastric irritation, as evidenced by lower ulcerative indices (2.45) compared to raw tacrolimus (6.73).

Controlled surface modification can enhance the efficacy of NS under in vivo circumstances, with the surface composition playing a crucial role in organ targeting. TPGS and Tween 80 were applied on the curcumin NS’s surface by physical adsorption [111]. Higher brain concentrations of curcumin were observed with Tween 80-coated NS compared to both the curcumin solution and TPGS-coated NS (*p* < 0.05). The enhanced brain delivery of Tween 80 coated NPs was attributed to the absorption of ApoE and/or ApoB from the blood, facilitating transport into the brain via receptor-mediated endocytosis. Although TPGS-coated NS also exhibited higher brain targeting than the curcumin solution (*p* < 0.05), TPGS-NS resulted in higher curcumin levels in the liver, spleen, and lungs.

Recent advances in NS technology include surface modifications to improve stability, targeted drug delivery for precision medicine, and innovations in preparation techniques like sonication and SCF methods. With their increasing application in clinical trials and the biopharmaceutical industry, NS represent an interesting approach for enhancing the therapeutic efficacy of many drugs. Table 2 summarizes the formulation composition, preparation methods, and key features of oral NS.

#### 5.1.1. Powders

Liquid NS face various physical and chemical stability problems, such as particle aggregation, sedimentation, and Ostwald Ripening along with microbial growth during storage. To address these problems, liquid NS were modified into solid forms through processes like lyophilization, spray-drying, or spray freeze-drying. Powders are mixtures of drugs and excipients that can be processed into more complex formulations such as tablets, capsules, and granules. NSs typically encounter stability challenges related to physical phenomena like Ostwald ripening and agglomeration and chemical reactions like hydrolysis. Therefore, pharmaceutical NS containing thermostable adjuvants can be transformed into dry powders using methods like spray-drying and fluidized bed drying, while techniques like freeze-drying and vacuum drying are more suitable for thermosensitive substances.

The drying process to create a compaction-ready powder or granule is crucial, as it can lead to undesirable solid characteristics and the potential growth of NPs. Indomethacin NS were created using wet media milling and dried by the fluidized bed technique using carriers including lactose, microcrystalline cellulose, and crospovidone, with varying binder contents while granulating [118]. The pellets were compressed into tablets, which showed an improved in vitro dissolution performance, especially with a higher binder content (PVP) and spray rates that minimized NP growth compared to raw indomethacin tablets.

In a recent study, poorly aqueous-soluble and poorly bioavailable albendazole was formulated into an NS using the twin centrifugation with Kollidon^®^ VA64 and sodium lauryl sulfate as the stabilizers [119]. The NPs, with a size of less than 300 nm, were coated with microcrystalline cellulose to form nanopellets and encapsulated in EUDRACAP^®^ with the objective of targeting the colon. Both the NS and granules demonstrated a substantial drug release (~60% and ~55%, respectively) at a colonic pH. The NS also showed strong anticancer activity, with IC_50_ values of 1.18 µM in HCT 116 and 3.59 µM in HT-29 colorectal cancer cells.

A study demonstrated the conversion of drug NS into stable, redispersible, inhalable nano-agglomerates using in situ thermal gelation and spray-drying [120]. Itraconazole NS were co-spray-dried with methylcellulose to provide superior thermal protection. Optimized spray-drying preserved the particle size with a near-unity Sf/Si ratio. Although various factors influenced the aerosol performance, no clear trends emerged. The nano-agglomerates exhibited an excellent in vitro aerosol performance, with fine particle fractions exceeding 50% and aerodynamic diameters ranging from 2 to 3 µm, making them well suited for deep lung delivery applications.

A key challenge in the process of modification into solid forms is ensuring that the NPs can redisperse effectively when mixed with water or gastric fluids. Redispersants such as sucrose, trehalose, lactose, maltodextrin, and mannitol play a crucial role in preserving the integrity of NPs when NS are dried into powders through diverse techniques [121]. Their primary function is to ensure that the dried NPs redisperse back to their original size upon reconstitution in water or biological fluids, preventing aggregation during the drying process. These sugars act as bulking agents to enable rapid redispersion forming while also providing cryoprotection during the freeze-drying process. Selecting the optimal redispersant based on the drying technique and drug properties is crucial for maintaining the particle size and stability, as well as preserving the enhanced dissolution, bioavailability, and stability of the drug [122]. Suitable lyophilization protectants/stabilizers include a variety of substances such as sugars (e.g., sucrose or trehalose), hydroxy alcohols (e.g., glycerol or sorbitol), amino acids (e.g., glycine or arginine), and mixtures of monosaccharides. Two main theories explain their protective mechanisms during lyophilization: the water substitution hypothesis and the glassy state hypothesis. The water substitution hypothesis suggests that these protectants form hydrogen bonds with proteins or the surfaces of NPs, replacing water molecules and stabilizing the structure during the drying process. During dehydration, sugars replace water, which stabilizes the distance between NPs and maintains the stability of the NS. In contrast, the glassy state hypothesis posits that as water is removed, the solution becomes highly concentrated and forms a noncrystalline, glassy state. This viscous environment surrounds proteins, preventing their movement and preserving their structure and properties while also protecting the NPs.

A recent study identified the optimal formulation for the lyophilization protectant consisting of oligomeric mannose (0.46% *w/v*), maltose (0.44% *w/v*), and sorbitol (0.05% *w/v*). Under these conditions, the lyophilized astaxanthin NP powder had a resoluble particle size of 472 nm, which is 1.32 times larger than the particle size before lyophilization. When the resultant powder was stirred with water, it formed a pink, fluffy coating that vanished entirely in ten seconds [123].

A study systematically assessed the impact of freeze-drying, processing conditions, and different cryoprotectants on cilostazol NS, with a primary focus on the particle size after redispersion [124]. The physical characteristics of formulations with trehalose (10%), maltodextrin (5%), or PEG 1500 (10%) were further investigated. The freeze-dried NCs successfully retained their original size and polymorphic form A, while significantly enhancing their dissolution rates (over 90% in 5 min) compared to untreated cilostazol (less than 30% for 60 min). Research has demonstrated that sucrose laurate is an effective stabilizer, allowing dried NCs of neutral drugs like fenofibrate, danazol, and probucol (150–300 nm) to disperse fully into their original sizes with mild agitation after drying by various methods [125]. The preliminary findings suggest sucrose laurate can also disperse acidic and basic drugs, indicating wide applicability. Similarly, lactose laurate, another stabilizer, performed well.

Hummer acoustic resonance technology (HART) is an advanced approach for NS preparation, enabling the efficient development and evaluation of multiple formulations with high throughput capabilities and excellent scalability for process optimization [126]. In a study, the optimal andrographolide NS was produced with a size of 223.99 ± 3.16 nm, PDI of 0.095 ± 0.007, and a ZP of –33.20 ± 0.58 mV, using PVP K30 and SDS as stabilizers. Significantly improved dissolution rates for NS and its solidified forms in both pH 1.2 and pH 6.8 buffers were reported as compared to the controls. In summary, HART offers a promising method for converting NS into solid powders, with benefits including precise control over the particle size and the preservation of sensitive drugs. However, its high energy consumption and complex scaling requirements may present challenges for large-scale applications.

Electrospray is a versatile technique used to produce powders from NS and surface coatings by applying an electric field to break the liquid into fine droplets, which then undergo solvent evaporation to form solid NPs [127]. This method allows for precise control over the particle size and morphology, which is essential for optimizing drug delivery. Electrospraying is particularly advantageous for converting liquid NS into solid forms, making them easier to handle and store, while also preserving the stability of heat-sensitive drugs due to its relatively low operating temperature. However, challenges include scaling up the process and optimizing parameters such as the solvent choice, voltage, and flow rate [128].

The electrospinning technique is an advanced process used in the preparation of NS, where an electric field is employed to produce fine polymer fibers or particles from a liquid solution or melt. This method enables the encapsulation of poorly water-soluble drugs within nanostructures, enhancing their solubility and bioavailability. It is particularly advantageous for its simplicity, scalability, controlled particle size and morphology, and ability to incorporate multiple therapeutic agents or excipients into the NS. A recent study demonstrated that polydopamine-coated paclitaxel-PEG NCs embedded in electrospun nanofibers enhanced the antitumor efficacy in a murine cervicovaginal tumor model. The nanofiber implant also achieved prolonged vaginal residence, improved transmucosal penetration, and minimal mucosal irritation [129].

#### 5.1.2. Pellets

Pellets, as multiparticulate dosage forms, provide numerous benefits, including a lower risk of dose dumping, shorter gastric residence time, and reduced dependence on gastric emptying. They also minimize intersubject variability in gastrointestinal transit times, enhance distribution to reduce local irritation, improve bioavailability, lower systemic toxicity risks, and offer more consistent pharmacokinetic behavior compared to traditional formulations. Additionally, pelletized NS offer several advantages, including excellent stability, good flow behavior, ease of dosing, low hygroscopicity, a high bulk density, and a dense, uniform surface that enhances their overall performance in various applications. Several pelletization techniques, including extrusion spheronization, powder layering, spray granulation, melt extrusion, and cryopelletization, can be used to convert NS into solid pellets, thereby improving their physical and chemical stability.

The hot melt extrusion (HME) method involves applying pressure and heat to melt or soften materials, which are subsequently extruded through a die to create uniformly shaped products. The advantages of the HME technique include the elimination of solvents and water, fewer processing steps that reduce the drying time, simplicity, continuous efficiency, the uniform dispersion of fine particles, and good stability under varying pHs and moisture conditions. HME is used for taste masking, enhancing the solubility of poorly soluble drugs, and preparing modified-release solid oral dosage forms. It is commonly employed to create amorphous solid dispersions that increase the dissolution rates, though they may face stability issues such as recrystallization during storage. Other key limitations of the HME method include high energy requirements, limited applicability to heat-sensitive materials due to the elevated temperatures, and the potential melting or softening of low melting binders during the handling and storage of the agglomerate. Dried amorphous clotrimazole NS were prepared by combining antisolvent precipitation with HME technology [130]. The NS and matrix forming microcrystalline cellulose were directly fed into the extruder using separate feeding devices, improving drug uniformity in the extrudate, which is crucial for low-dose formulations. Key process factors like the feed rate and input temperature were found to influence the product’s redispersibility and moisture content. A moderate inlet temperature effectively removed the residual moisture without affecting the redispersibility of the dried NS. The drug remained amorphous post extrusion, as confirmed by DSC and XRPD, while polarized light microscopy was ineffective due to the presence of the semicrystalline nature of microcrystalline cellulose. It is important to note that the coexistence of crystalline and amorphous forms can be assessed using suspended-state NMR and Raman spectroscopies. In vitro dissolution studies showed enhanced solubility and dissolution, linked to the amorphous nature of the nanosized drug particles. A comparative evaluation of the impact of the matrix surface area of solid dispersions with hydrophilic polymers via the nanoextrusion technique and amorphous solid dispersions in enhancing the dissolution rate of a potent poorly aqueous-soluble model drug, griseofulvin, has been investigated [131]. While fine grinding was necessary for the extrudates to achieve an immediate release, coarse milling was sufficient for the nanocomposite to rapidly release low-dose drugs. Lastly, this work suggests that drug loading, drug release, and the specific surface area of the milled dispersions interact in a complex way.

The advantages of extrusion spheronization include a uniform pellet size, controlled release properties, and suitability for both low- and high-dose formulations. Limitations include the need for specialized equipment, a time-consuming process, and challenges in scaling up for large-scale manufacturing. Wet-milled and normal suspensions were added to solid carriers made from microcrystalline cellulose, isomalt, and crospovidone to create matrix pellets via the extrusion/spheronization technique [132]. The NPs were successfully reconstituted from the solid dosage forms, resulting in a decrease in particle size and a significant enhancement in the thermodynamic solubility and the dissolution rate of cilostazol in gastric media in comparison to pure surfactant dispersions. Differential scanning calorimetry and XRD confirmed that cilostazol transitioned from Form A to an amorphous state as a result of the extrusion process.

Simvastatin NS were prepared using 7% HPMC (stabilizer), 0.03% butylated hydroxyanisole (antioxidant), and 0.2% citric acid (synergistic antioxidant action) via low-temperature grinding [133]. Drug and SDS in a 1:5 ratio were uniformly dispersed and applied to sugar pellets using a fluid bed granulator. The NS had an average particle size of 0.74 µm, with 80.6% of the particles being smaller than 1 µm. The relative bioavailability of the drug from the NS’s layered pellets was higher (probably due to a greater dissolution rate compared to a commercial tablet).

#### 5.1.3. Tablets

Pharmaceutical tablets are solid dosage forms manufactured through compression or molding, typically using granules combined with appropriate excipients. NS can be transformed into dry powders by various techniques as well as stabilizers, as described in Section 5.1.1. For instance, naproxen granules prepared from a nanodispersion using spray-drying were compressed into tablets using mannitol, which acted as a bulking agent, stabilizer, and disintegrant. These tablets exhibited rapid dissolution, completely dissolving within one minute under both sink and nonsink conditions [134]. Similarly, lovastatin NCs were developed through spray-drying using PVP K17 (20%) and sodium lauryl sulfate (5%) as the stabilizers. The resulting sustained release tablets were optimized using lactose as a diluent, Avicel PH101 as a compression aid, and Ac-Di-Sol as a superdisintegrant [135].

Spray-drying is a versatile, single-step, continuous process that transforms liquid feeds into dried particles, making it an effective platform for particle and crystal engineering. While spray-drying offers a straightforward method for converting NS into powders, its limitations include the risk of degradation of heat-sensitive drugs, the potential for particle agglomeration, high energy consumption, and challenges in controlling particle morphology. These limitations need to be considered carefully when using spray-drying for the preparation of pharmaceutical NS. The effect of dispersants on the dissolution of poorly aqueous-soluble itraconazole nanocomposites prepared using spray-drying techniques was studied [136]. Superdisintegrants like sodium starch glycolate, crospovidone, and croscarmellose sodium, co-milled with itraconazole stabilized by HPC, demonstrated faster dissolution compared to sugars like mannitol and sucrose used as matrix formers. A correlation between the dispersants’ efficacy and swelling capacity points to a mechanism of erosion or disintegration brought on by swelling. This approach enabled the formulation of nanocomposites with a high drug-loading capacity (>60% *w*/*w*) and immediate drug release. Another study demonstrated the superior dissolution rate of risperidone spray-dried NS formulated into orally disintegrating tablets compared to commercially available products [137]. Additionally, research has explored converting PLGA polymeric NP suspensions into solid dosage forms using fluid-bed processing. It was noted that dried intermediates containing polymeric NPs could successfully redisperse into their original nanoparticulate form after dispersion [138]. Key factors such as the NP concentration in the granulation suspension and the ratio of the spraying rate to the atomization air pressure were found to critically influence the redispersibility and physicochemical properties of the granules.

A successful spray-dried NS formulation depends on the drying temperature, matrix-former type and content, and particle size. NS of naproxen and itraconazole, stabilized with Kollidon^®^ VA 64 and sodium lauryl sulfate, were spray-dried using lactose, trehalose, or sucrose as the matrix formers [139]. The results revealed that the outlet temperature and drug content significantly influenced the redispersibility of the NS. The maximum achievable drug content for a redispersible product depended on the outlet temperature, the matrix former’s glass transition temperature, its proportion, and the NS’s particle size. Agomelatine NS were prepared using wet media milling using HPC as the stabilizer and subsequently solidified through spray-drying. A polymorphic change from agomelatine form I to form II occurred during the milling process. Crystal lattice simulations indicate that form I crystals exhibit high mechanical anisotropy, which may contribute to their rapid particle size reduction before undergoing polymorphic transformation. The spray-dried NS was then processed into mini tablets, which demonstrated significantly faster dissolution compared to physical mixtures of agomelatine [60]. In another study, celecoxib was formulated into NS via precipitation and HPH, followed by spray-drying or freeze-drying [140]. The choice of solvent, stabilizer, and surfactant significantly impacted the NP crystallization, size, and solubility. Both drying methods preserved the drug’s chemical stability and enhanced the dissolution rates compared to the raw drug. However, freeze-dried NS showed a brief initial delay in dissolution, followed by a lag phase due to particle aggregation.

Freeze-drying is a valuable pharmaceutical batch process for removing solvents, particularly water, while preserving the product integrity. It enhances the stability of thermolabile drugs, particularly for long-term storage, produces highly porous products with a low moisture content, and enables the preparation of reconstitutable NS. While effective, it is time consuming, energy intensive, and costly, which limits its scalability. The freeze-drying process can destabilize NPs due to freezing and desiccation stresses, where phase separation during freezing results in ice and a cryo-concentrated suspension with a high concentration of NPs, causing particle agglomeration. Ice crystallization can also exert mechanical stress, further impacting NP stability. To prevent product collapse, primary drying should occur below the collapsed temperature, and cryoprotectants should be added before lyophilization to mitigate these stability challenges [141]. A study was conducted to evaluate the impact of freeze-drying with different cryoprotectants, such as TPGS and folate-modified distearoylphosphatidyl ethanolamine-PEG, on the physicochemical properties of resveratrol and quercetin NS [142]. NS formulated using the antisolvent precipitation method exhibited resveratrol and quercetin NPs measuring approximately 210 nm and 110 nm, respectively. The dissolution rate of the drug NPs increased by 6–8-fold and exhibited long-term stability. Silymarin, which has limited water solubility and oral bioavailability, was formulated into lyophilized NS tablets using a sonoprecipitation technique accompanied by freeze-drying [143]. PVA served as a stabilizer and binder, while mannitol functioned as a cryoprotectant and disintegrant. The optimized formulation displayed a porous structure, rapid disintegration, friability below 1%, and enhanced solubility and dissolution rate. The active freezing technique involves closely monitoring and regulating the freezing process to achieve a specific freezing rate or temperature profile. The method is used to convert metastable NS into redispersible nanocrystalline powders that are ideal for oral drug delivery [144].

#### 5.1.4. Capsules

Hard gelatin capsules offer multiple benefits, including ease of formulation, as they can accommodate various drug types like powders, granules, and even NS. For example, NS-loaded capsules of Novartis compound A and itraconazole demonstrated enhanced bioavailability in animal studies. The gelatin shell dissolves rapidly, ensuring the quick release and absorption of the drug. Capsules also mask unpleasant tastes and odors, improving patient compliance, and come in various sizes, providing flexibility in dosing. Additionally, they offer tamper evidence for added security and ensure precision and uniformity in dosing. Many patients find capsules easier to swallow than tablets, making them a preferred choice for oral medications.

A study optimized *Kaempferia parviflora* NS to enhance intestinal absorption using antisolvent precipitation [145]. The optimal formulation used sodium lauryl sulfate (3%), achieving stable particle sizes (100–300 nm) with high stability over one month. The NS encapsulated in a hard gelatin capsule showed rapid dissolution (over 80% within 30 min) and increased intestinal absorption by about 10-fold, demonstrating its effectiveness for improved bioavailability.

#### 5.1.5. Film

Buccal films adhere firmly to the oromucosal membrane to provide precise dosing and enhanced drug absorption, making them ideal for both local and systemic treatments [146,147,148]. These films are particularly suited for buccal use due to their versatility, comfort, flexibility, lightweight nature, adaptability, and resilience under mechanical stress, as well as their customizable size [147,149]. Integrating NS into films offers several benefits including a minimized premature or excessive drug release, and decreased fluctuations in plasma levels and interpatient variability. NS, particularly NCs, can accommodate a variety of hydrophobic drugs for sustained release. A three-layer buccal film containing carvedilol NS was developed, featuring an outer mucoadhesive layer, an NS core, and an innermost backing membrane [145]. The NS had a mean particle size of 495 nm, a negative ZP of −17.21 mV, and a PDI of 0.203. When added to an HPMC and carbopol hydrogel layer, this film significantly enhanced the bioavailability (916%) and achieved a 7.3-fold higher Cmax and Tmax of 4 h compared to tablets, mainly due to the nanosized particles. Another study developed and evaluated oral mucoadhesive films containing clotrimazole NS to treat oral candidiasis [150]. The clotrimazole NS were formulated using a bottom-up method with a surfactant, benzyl succinyl chitosan, and incorporated into a film made from catechol-functionalized hyaluronic acid and PVA. The films released clotrimazole slowly, achieving complete release within 6 h, and were nontoxic to normal cells while demonstrating significant antifungal activity compared to a traditional clotrimazole suspension.

The primary aim of orodispersible film is to dissolve or disintegrate rapidly in the oral cavity, forming a solution or suspension that is subsequently swallowed for absorption [146]. Researchers improved the bioavailability of olmesartan medoxomil by formulating it as an NS using an antisolvent precipitation ultrasonication method, resulting in NPs with a size of 120 nm and ZP of −45 mV [151]. The optimized fast-dissolving film showed rapid disintegration (20.50 s) and achieved high drug dissolution rates (87.53% in 6 min and 95.99% in 10 min). Compared to standard tablets, the fast-dissolving tablets substantially increased the bioavailability (209.28%), Cmax (from 66.62 to 179.28 ng/mL), and AUC_0–72_ (from 498.36 to 1083.67 ng h/mL).

The ginkgolide B NS lyophilized powder-based orodispersible film was developed using solvent casting, with its formulation optimized through single-factor and orthogonal tests [152]. The NS, prepared via media milling, was lyophilized with mannitol as a lyoprotectant. The final film, composed of NS (35.6%), PVA (49.4%), PEG 400 (10.7%), and sodium carboxymethyl starch (4.3%), exhibited ideal properties, including a rapid disintegration time of ~30 s and consistent particle sizes (~210 nm in a reconstituted form). It maintained stability and uniformity for 30 days. An electron microscopy image showed a smooth surface with evenly distributed particles (~200 nm), XRD analysis indicated reduced crystallinity, and in vitro testing revealed complete dissolution within 10 min.

A fast-dissolving orodispersible film incorporating nitrendipine NPs has been reported [153]. Drug NS were prepared using the antisolvent sonoprecipitation method and subsequently processed into films through solvent casting. The optimized NS achieved a particle size of ~500 nm and significantly improved solubility (~26 times than pure drug). The film exhibited desirable properties, including thickness (0.148 mm), tensile strength (8.25 kg/cm^2^), and a rapid disintegration time (24.60 s). Dissolution studies showed a complete drug release (8 min from lyophilized NCs and 3.5 min from film) compared to 30 min for conventional tablets. Pharmacokinetic tests in rabbits demonstrated higher bioavailability from films than from tablets.

The improper drying of drug NPs can cause irreversible aggregation, decreasing the dissolution rate. Loading drug NS onto films offers a simple and efficient solution to this issue. An optimized fast-dissolving oral film with paroxetine NS was developed using the solvent casting method with the aid of a full factorial design [154]. The optimized film demonstrated strong mechanical properties, a short disintegration time (17 s), and greater dissolution (96.02% in 10 min). Permeation studies using the chicken buccal model showed over threefold increased drug absorption compared to the pure drug. Clinical testing in healthy volunteers confirmed the improvement in the relative bioavailability (178.43%) in comparison to a marketed tablet, highlighting the effectiveness of such a formulation in enhancing drug bioavailability.

The 3D printing of pharmaceuticals enables the creation of customized drug delivery systems, such as tablets and films, with tailored shapes, sizes, and release profiles. Incorporating NS into these films enhances the solubility and bioavailability of poorly soluble drugs by increasing the surface area and dissolution rates [155]. This approach allows precise control over drug loading, release, and personalized dosing, offering flexibility for combining multiple drugs or excipients. A rapidly dissolving oral film loaded with indomethacin NCs was developed using a semi-solid extrusion 3D printing method [156]. HPMC served as the film-forming polymer, glycerol as the plasticizer, and Poloxamer F68 as the stabilizer, producing particles with a size of 230 nm and PDI values below 0.20. NCs maintained their size range (300–500 nm) in all formulations. Films prepared with optimal polymer concentrations (2.85% and 3.5% *w/v*) were flexible, homogeneous, and disintegrated within 1–2.5 min, achieving complete drug release in 23 min. Additionally, a solvent-free, low-temperature 3D printing technique, termed melting solidification printing, was used to embed albendazole NCs in printlets [157]. These printlets demonstrated improved dissolution rates compared to spray-dried NCs, with physical and chemical stability maintained for six months during storage. A comparison illustrating the conversion of NS to various formulation types, along with their advantages, limitations, preparation methods, and typical applications, is summarized in Table 3.

### 5.2. Ophthalmic

Conventional ophthalmic products face low ocular bioavailability due to anatomical, physiological, and biochemical barriers, and the physicochemical properties of the drug. With their smaller particle size and improved mucoadhesion, NS stay longer in the eye’s cul-de-sac, providing sustained release and avoiding frequent dosing. The growing use of NS in ocular drug delivery offers new possibilities for overcoming the limitations of conventional ophthalmic formulations, potentially improving treatment outcomes for conditions like infections and inflammation. For instance, voriconazole NS prepared using the emulsion solvent evaporation technique with Eudragit^®^ RS 100 as a stabilizer and N-methyl-2-pyrrolidone as a permeation enhancer demonstrated excellent ocular bioavailability in treating fungal keratitis caused by *Candida albicans* [85]. These findings emphasize the potential of NS as an effective platform for delivering poorly water-soluble drugs, including immunosuppressants, for the treatment of various ocular conditions. Utilizing advanced technologies can address stability challenges in ocular NS, resulting in more effective and dependable treatments for eye diseases [158]. Table 4 lists NS formulations developed for various ocular conditions along with their attributes.

A mucoadhesive gellan gum-based in situ gelling NS of posaconazole has been developed to enhance the contact time in ocular tissues [165]. The NS was formulated using microfluidization and optimized utilizing QBD principles with gellan gum (0.4% *w/v*) to provide adequate viscosity and mucoadhesiveness. The NS exhibited a larger zone of inhibition (~15 mm) compared to the marketed itraconazole NS (~11 mm). It was determined to be nonirritant, receiving a potential irritancy score of 0.85. Furthermore, the NS achieved a higher drug release rate (~35%) compared to a coarse suspension (~10%), and permeation studies in goat corneas showed that around 70% of the drug was retained in the membrane. NS of moxifloxacin and pamoic acid, designed for mucus penetration, showed superior efficacy against *Staphylococcus aureus* in bacterial keratitis with once daily dosing, outperforming standard eye drops administered thrice daily [166]. Pharmacokinetic studies indicated effective drug distribution in anterior ocular tissues.

Unlike conventional suspensions, which may contain larger particles that cause irritation, NS minimize discomfort, enhance patient comfort, and require the minimal use of potentially harmful solvents [59]. They also avoid the high osmolarity associated with some ophthalmic solutions, reducing potential damage to ocular tissues. Additionally, NS increase the saturation solubility of poorly water-soluble drugs, making them more effective in lachrymal fluids where conventional suspensions fall short. NS offer formulation versatility as they can be combined with hydrogels, ointments, or mucoadhesive bases to achieve tailored release profiles and extend residence times. Moreover, certain formulations containing Eudragit polymers help to stabilize sensitive drugs, prolonging their shelf life and effectiveness. These advantages make ocular NS particularly beneficial for treating chronic eye conditions and infections, where a sustained release and minimal irritation are crucial for successful treatment outcomes. Eudragit RS 100 was used as a polymer to optimize and enhance the intraocular delivery of itraconazole NS prepared via solvent evaporation [167]. The data demonstrated that the optimized NS possess moderate particle sizes (332.7 to 779.2 nm), ZP (+0.609 to 16.3 mV), and entrapment efficiency (61.32% to 76.34%). Additionally, higher corneal permeation and antifungal efficacy against *Candida albicans* and *Aspergillus flavus* were shown by NS when compared to a marketed formulation and an itraconazole eye drop formulated with sulfobutyl ether-β-cyclodextrin.

Carbon dots, spherical NPs under 10 nm in size, are particularly effective due to their strong fluorescence, high water solubility, small size, ease of synthesis and modification, low toxicity, and good biocompatibility [168,169]. A composite ocular drug delivery system was developed for the topical administration of diclofenac, utilizing carbon dots synthesized through a one-step hydrothermal method from hyaluronic acid and carboxymethyl chitosan [170]. These carbon dots were incorporated into a thermosensitive in situ gel made of poloxamer 407 and 188, creating a gel with sustained release capabilities for 12 h. Ex vivo fluorescence studies demonstrated that the gel enhances bioimaging and tracing in ocular tissues, with a 3.45-fold increase in drug bioavailability in the aqueous humor (compared to eye drops), indicating prolonged corneal retention.

Advancements in drug-loaded contact lenses have transformed ocular drug delivery by enhancing both efficacy and convenience over traditional eye drops [171]. These lenses are designed with embedded drug reservoirs or NPs that gradually release therapeutic agents over extended periods, improving treatment adherence and reducing side effects. Recent innovations include hydrogel-based lenses and silicone hydrogel materials that support longer wear and precise drug dosing, making them promising for chronic conditions like glaucoma, dry eyes, and infections. On the other hand, these lenses often provide only a short release duration, which may not be sufficient for long-term treatment needs [171]. Additionally, they can face issues with storage stability, as some drugs may degrade over time when stored in the lenses. A novel contact lens with a pH-sensitive inner layer provides an extended, stable drug release for ocular therapy [172]. Fabricated with ethyl cellulose, Eudragit S100, and polyhydroxyethylmethacrylate hydrogel, the lens achieves an optimized daily release of diclofenac and can be stored with minimal drug loss. In vivo studies indicate sustained drug delivery for over 24 h, making it a promising option for treating various eye conditions.

Intravitreal injections can effectively deliver small molecules (less than 500 Da) directly to the vitreous humor. However, the repeated use of this route for drug administration may lead to complications related to the retina and an increase in the intraocular pressure [173]. Research was undertaken to develop a hybrid system combining NS and DMNs as a minimally invasive alternative for the trans-scleral delivery of triamcinolone acetonide NS with a particle size of 246.65 ± 8.55 nm [174]. The NS-loaded DMNs were capable of piercing excised porcine sclera, achieving insertion depths of over 80% of the needle height and dissolving rapidly (less than 3 min), while plain-drug-loaded DMNs took more than 8 min to dissolve. Trans-scleral deposition studies indicated that the NS-loaded DMNs deposited 56.46 ± 7.76 μg/mm^2^ of the drug in the sclera after 5 min, a 4.5-fold increase compared to the plain-drug-loaded DMNs. The drug NS containing the DMN array demonstrated biocompatibility with ocular tissues when evaluated using the hen’s egg chorioallantoic membrane assay and cytotoxicity. Overall, this hybrid system of NS and DMNs offers a promising, minimally invasive approach to treating retinal diseases effectively.

NS are prone to instability, often resulting in agglomeration or sedimentation that reduces their efficacy. While stabilizers and surface modifiers are typically used, they can introduce toxicity and complicate regulatory approval. Researchers are investigating the use of advanced materials like biodegradable polymers and lipid-based carriers to improve long-term stability while ensuring biocompatibility and safety [175]. In this context, stimuli-sensitive drug delivery systems that respond to pH, temperature, and enzyme activity appear to be interesting solutions to ocular NS stability issues. Microfluidics and 3D printing are promising techniques for creating stable, finely designed NS. Microfluidics provide precise NP production, lowering the possibility of aggregation, whereas 3D printing enables customized drug delivery systems, improving the ocular dosage accuracy and facilitating patient-specific therapies. The primary distinctions between microfluidizers and conventional HPH lie in the type of pump utilized, the pressure levels they can achieve, and the presence or absence of a nozzle [71]. A study focused on enhancing the stability of iodine NS (^127^I) through nanoprecipitation using microfluidic devices and examining the influence of preparation parameters on their stability [176]. Artificial neural networks were employed to optimize the process, analyzing the relationships between the input variables (solvent temperature, antisolvent flow rate, and solvent flow rate) and output parameters (sedimentation time and PDI) across the test samples. The optimization revealed that the microfluidic preparation parameters significantly impacted the stability of the NS.

### 5.3. Transdermal

Transdermal drug delivery encounters significant formulation challenges, particularly due to the existence of intercellular lipids within the stratum corneum (SC), which form a strong permeability barrier, as well as the physical barrier provided by keratinocytes [177]. However, small lipophilic molecules and certain hydrophilic or polar compounds can penetrate through the living epidermis and finally into the dermis, where there are blood capillaries. The physicochemical properties of a drug, governed by factors such as the molecular size, concentration, and solubility, are critical for successful transdermal delivery [178]. According to Lipinski’s Rule of Five, drugs with a molecular weight under 500 Da, low polarity, and high lipophilicity are more likely to reach the dermis. The Stokes–Einstein equation further highlights the importance of molecular size and diffusion, as smaller molecules tend to diffuse more easily through the compact structure of the SC [179,180]. In addition, the nonionic state of NS at the site of application (i.e., SC with pH 4.2 to 5.6) can improve drug penetration [181,182]. Additionally, factors such as the application area, method, duration, skin age, and the use of carriers that modify the skin’s barrier function play a role in the overall effectiveness of transdermal therapy [179].

NS-based transdermal delivery received much attention after the commercialization of cosmetic products of rutin (Juvedical^®^) [183]. Two possible mechanisms for the transdermal delivery of NS are suggested: a) an increased transdermal concentration gradient and b) extended retention on the skin’s surface to sustain the concentration gradient [183]. The transdermal drug delivery of NS can occur through multiple pathways, including intracellular and intercellular penetration, as well as delivery via hair follicles, sweat glands, or sebaceous glands [184]. For intracellular penetration through SC cells, the drug must exhibit favorable physicochemical properties such as optimal solubility, a distribution coefficient, drug concentration, cell membrane permeability, and the chosen mode of administration. The particle size of the NS significantly influences the efficiency and pathway of the transdermal transport. For example, a study comparing curcumin NPs of 140 nm, 400 nm, and 730 nm demonstrated that smaller particle sizes can enhance the penetration efficiency, highlighting the critical role of nanoscale dimensions in optimizing transdermal delivery. The 400 nm NS had a higher steady-state flux, but their cumulative penetration was lower than that of the 140 nm and 730 nm formulations [183]. It was noticed that the curcumin molecules dissolved in the SC were rapidly distributed throughout the skin and hair follicles, whereas intact NS did not penetrate the skin. The NS were found to more easily accumulate in hair follicles due to their large openings, ranging from 10 to 210 μm in diameter.

Microneedles bypass the skin’s barrier, allowing the efficient administration of various therapeutics, including vaccines and peptides. Once inserted into the skin, DMNs dissolve, releasing the embedded drug or therapeutic agent into the body. It was proposed that drug NPs accumulated as a depot using DMNs could quickly dissolve in the skin’s interstitial fluid and subsequently be absorbed into the rich dermal microcirculation to achieve the desired plasma drug concentration levels [185]. The successful integration of drug-loaded NS into DMNs for transdermal delivery has been reported [186]. In this study, cholecalciferol NS were developed via sonoprecipitation to improve transdermal delivery by incorporating them into mechanically strong DMNs using a centrifugation-assisted micromolding method. The drug NS, stabilized with PVA, achieved a particle size of ~300 nm and demonstrated good mechanical strength when coupled with PVA. The DMNs penetrated approximately 375 μm into the skin model (Parafilm M^®^). A permeation study using porcine skin showed that drug-NS-loaded DMNs significantly enhanced skin permeation (498.19 ± 89.3 μg) compared to drug NS patches without microneedles (73.2 ± 26.5 μg) over 24 h. Promising results were reported with other drugs like itraconazole [187], metronidazole [188], baclofen [189], and ibuprofen [190], highlighting the effectiveness of NS and microneedle technology in improving cutaneous drug delivery. Selected examples of NS formulations for transdermal delivery, detailing the active ingredient, formulation technique, and key highlights, are tabulated (Table 5).

### 5.4. Pulmonary

Pulmonary drug delivery systems administer medications directly into the lungs through inhalation, making them highly effective for treating respiratory diseases such as asthma and chronic obstructive pulmonary disease. Key advantages include targeted delivery, rapid absorption via the alveoli’s large surface area, and lower systemic exposure, which reduces the side effects. However, these systems can be device-dependent, with efficacy influenced by proper inhaler use and patient inhalation patterns. For instance, conventional aerosols face several challenges, and particle agglomeration and aggregation lead to deposition in the pharynx and upper respiratory tract [198]. The passive or active targeted administration of a drug to a specific tissue, either an intracellular compartment or cell, by managing release kinetics and shielding the therapeutic agents improves its therapeutic index by enhancing its specificity [199]. Furthermore, the location of aerosol accumulation in the respiratory system is controlled by the aerosol’s physical qualities, inhalation circumstances, and the structure of the respiratory airways [200].

The human lung has several mechanisms such as mucociliary clearance, mechanical clearance, alveolar macrophages, and enzymatic degradation in various regions which limits the diffusion and deposition of aerosols in the lung [201]. While these mechanisms safeguard the respiratory tract from harmful exposure to foreign substances, they also present significant challenges for administering drugs via inhalation [202]. Recent research on oropharyngeal drug deposition has revealed additional factors influencing transport and deposition, such as the particle velocity, mouthpiece diameter, and electrostatic effects associated with the delivery device [198]. Apart from impaction, sedimentation, and diffusion, the fundamental deposition mechanisms depend mainly on the size of the inhaled particles and their inhaling rate [203]. The pulmonary delivery of medications via aerosol is mainly facilitated by different inhalation devices (nebulizers, metered dose inhalers, dry powder inhalers, and soft mist inhalers), categorized according to the physical state [204].

Targeted NP delivery to the lungs is an emerging field, offering the advantages of a reduced dosage and minimized side effects by limiting systemic exposure. NS offer significant benefits in pulmonary drug delivery [205]. They enhance the bioavailability of poorly soluble drugs, extend the retention time by adhering to the lung’s mucus layer, and allow for reduced dosages by concentrating the drug in the lungs. Recent advances in this field include NP-based inhalers with improved aerosol properties, dry powder NS for better stability, and functionalized NPs targeting specific lung cells. These advancements have enormous potential to enhance drug solubility, deliver drugs precisely, and reduce adverse effects in pulmonary therapy. The main physical parameters affecting the overall and regional deposition of inhaled chemicals in the respiratory tract are aerodynamic particle size distribution and particle velocity [206]. It is common practice to use the mass median aerodynamic diameter to assess the mass distribution of aerosol particles. For the fine particle fraction, which represents the percentage of particles with an aerodynamic diameter of less than 5 μm, the optimal size for lung deposition is calculated by the ratio of the fine particle dose to the delivered dose [207]. To achieve a respirable particle size and maximize the shelf life, excipients like mannitol, sucrose, sodium chloride, and trehalose are often incorporated [208]. Several biopharmaceutical products, predominantly protein-based, are currently approved in the United States and Europe for treating various diseases [209]. Given the large size of biopharmaceutical molecules, absorption enhancers are needed for their effective uptake at the alveolar level, although these enhancers may increase the particle size due to entrapment or encapsulation. It is crucial to control both the aerodynamic size and surface properties of the particles, as micronization can increase the particle charge and aggregation risk. Additionally, the rapid elimination of proteins from the lungs necessitates frequent dosing, which can affect patient compliance [210].

The drug’s nanoparticulate form allows for a quicker onset of action since it diffuses and dissolves more quickly in alveolar fluids. Additionally, the nano-products can prolong the drug release because of their enhanced affinity for mucosal surfaces. The NPs’ unique physicochemical properties, including uniform and narrow size distribution, make uneven drug distribution and delivery to the lungs unlikely compared to aerosols. When comparing hydrophobic budesonide NS to coarse and micronized drug particles, the lung distribution rate of the former was substantially higher (872.9 ng/g) and distinct (*p* < 0.05) [211].

Spray-freeze-drying is an effective approach for converting NS into dry powders and is particularly suitable for producing low-density porous particles for inhalation drug delivery [212]. In this process, active agents and excipients are co-sprayed into liquid nitrogen, quickly freezing the droplets, which are then freeze-dried into powders. Cefixime NS were solidified by spray-freeze-drying to produce inhalable microparticles [213]. The fine particle fraction values varied from 18.96% to 79.28%, with the highest achieved using trehalose at a 1:1 NP/carrier ratio and 20% leucine. Particle sizes ranged between 5.24 and 10.17 μm, with mannitol formulations having the broadest distribution and trehalose the narrowest. Most particles were spherical with varying porosity and no needle-like structures. The release rates for the selected formulations were 89.33% and 93.54% within the first 10 min, respectively. Recent examples of NS used in pulmonary drug delivery and their highlights are illustrated in Table 6.

### 5.5. Parenteral

The parenteral administration, especially via the intravenous route, is often preferable for quick action, bypassing the first-pass liver metabolism, and delivering drugs directly to the target site. However, developing injectable products for poorly aqueous-soluble drugs presents major challenges. Techniques currently used in increasing the drug solubility are by making salts or by adding co-solvents, surfactants, or cyclodextrin complexes, but they have limitations such as incomplete solubilization, hypersensitivity, toxicity, and restricted applicability to certain molecules. Additionally, the limited concentration of these agents as mandated by regulatory authorities presents a formidable challenge for formulation development scientists. On the other hand, the NS, along with other carriers, can be an option for drug targeting to various organs [220,221]. The targeting potential of nevirapine was improved by developing NS and surface-modifying them with serum albumin, polysaccharide, and PEG [222]. NS, which contain NCs of poorly soluble drugs suspended in an aqueous medium with a restricted concentration of stabilizers, are emerging as a promising solution for this issue. Parenteral NS are a widely used formulation approach in drug development, particularly when evaluating the safety of a new chemical entity in experimental animals during dose escalation studies [223].

Parenteral NS delivery utilizing NCs reduces the toxicity of nonaqueous formulations while enabling targeted drug delivery, making them valuable for precision therapies [224]. Parenteral NS offer advantages such as the enhanced solubility of poorly aqueous-soluble drugs and improved bioavailability of BCS class IIa drugs with high doses and BCS class IIb drugs with limited solubility and high melting points [28]. In general, intravenous NS are a promising option for high-dose, poorly soluble drugs [21]. Particles are typically stabilized by agents such as polymers or surfactants, but selecting safe and effective stabilizers is crucial for parenteral use due to concerns over toxicity and microbial risks. Moreover, the concentration of stabilizers beyond the critical flocculation concentration and critical micellar concentration may result in micellar solubilization leading to Ostwald ripening and system destabilization [225]. NS with particle sizes between 100 and 300 nm are ideal for utilizing the enhanced permeability and retention effect, beneficial particularly in solid tumors and hence for reduced toxicity [226]. Nanocrystalline suspensions have become a promising approach for developing parenteral prolonged release systems for insoluble drugs [227]. Crystalline transformation methods, including solid–solid transformations, solution-mediated transformations, transformations through raw material melting, and those from drug solutions, are often to produce stable crystalline pharmaceutical solids [228]. A uniform-sized nanocrystalline suspension-based montelukast parenteral prolonged-release delivery system was developed using the bead milling technique, utilizing polysorbate 80 as a suspending agent [4]. In rats, the NS with 200 nm and 500 nm particle sizes showed a longer pharmacokinetic profile for up to 4 weeks, with greater plasma drug concentrations than the injectable suspensions with 3 μm particle sizes. According to the histopathological investigation, the generated NS resulted in persistent granulomatous inflammation at the injection site, which disappeared after four weeks. Formulation challenges include the risk of aggregation, which can block intravenous lines and fine capillaries, which can potentially cause serious complications such as thrombus, embolism, or stroke. For example, particles larger than 7 μm and larger agglomerates can lead to a pulmonary embolism and pose a risk to patient safety. In recent years, parenteral NS use has been observed in toxicological and clinical formulations [229].

Parenteral NS require the careful consideration of key parameters to ensure safety and efficacy. Isotonicity and pH are adjusted for physiological compatibility, while particle size and ZP affect stability and drug delivery. Lyophilized formulations need to be easily redispersed, and sterility is ensured through filtration or aseptic processing. Multi-dose formulations include antimicrobial preservatives, and viscosity is controlled for easy administration. Stability studies ensure the formulation remains effective over time, and drug release profiles determine how the drug is released in the body. These factors are critical in developing an optimal NS for parenteral use. In addition, parenteral NS must meet stringent regulatory requirements, including QBD and current good manufacturing practice standards to ensure safety, quality, sterility, and stability. The FDA’s guidance for drug products containing nanomaterials outlines considerations for managing risks associated with these materials, emphasizing that the same safety, efficacy, and quality standards apply to nanomaterial-based products as to other drugs. Nanomaterials can be sensitive to process conditions and may face stability issues, such as changes in size or aggregation. Despite these challenges, drug developers must adhere to current good manufacturing practices, thoroughly assess CQAs, and evaluate the safety, efficacy, and quality of these nanomaterial based-drug products.

Amino-acid-derived copolymers such as albumin, lysine, leucine, and transferrin and synthetic nonionic polymers such as poloxamer 188 (Pluronics F68) and poloxamer 407 (Pluronics F127) are widely regarded as safe for parenteral applications by FDA. A comparative study of stabilizing excipients revealed that polysorbate 80-based NS caused stronger inflammation and slower recovery in male rats, while TPGS-based formulations showed a moderate response, combining features of both the polysorbate and poloxamer [230]. Soluplus, a polyvinyl caprolactam-polyvinyl acetate-PEG graft copolymer, serves as a solubilizer for poorly water-soluble drugs in parenteral formulations at moderate concentrations. It is compatible with various processes like spray-drying, high shear milling, solvent evaporation, and electrospinning. Its high flowability and controlled extrudability make it an effective stabilizer in pharmaceuticals [231]. Additionally, below its critical micelle concentration, Soluplus inhibits drug precipitation by preventing nucleation and crystal growth, while providing steric stabilization in a supersaturated state [232]. Similar to coarse suspensions, intravenous nanocrystalline suspensions can be categorized into two types: (a) pre-mixed and (b) dry powder NS for reconstitution.

Parenteral NS can be prepared through the bottom-up technique, which forms NPs by dissolving the drug in an organic solvent and using an antisolvent to precipitate the drug with a stabilizer. Common methods include SCF processes, sonocrystallization, spray-drying and controlled crystallization using freeze-drying [56]. In the top-down wet media milling technique, highly cross-linked polystyrene beads are commonly used as the milling media to minimize cracks and abrasions. For example, the commercial technology NanoCrystal^®^ utilizes PolyMill media made from polystyrene beads to prepare NS meant for parenteral use [28]. Aseptic processing, autoclaving, or gamma radiation (for dry powders) are the three methods used to sterilize injectables. Typically, the active pharmaceutical ingredient and excipients are separate, and then combined under aseptic conditions. Alternatively, the excipients can be sterilized in solution and mixed with the drug aseptically. The sterile filtration of aqueous NS is another option, as demonstrated by NanoCrystal™ for the X-ray contrast agent iodipamide, which used a 0.2 μm Supor^®^ filter to retain 100% of *Pseudomonas diminuta*.

Nanocrystalline suspensions can be utilized for drug targeting owing to their surface potential and in vivo behavior. After an intravenous injection, drug NCs can passively target tumors via the enhanced permeability and retention effect or be absorbed by macrophages if dissolution is delayed [233]. This leads to a prolonged release, reducing toxicity while maintaining drug efficacy, particularly beneficial for certain drugs like antineoplastics [234]. Differing pharmacokinetics were observed between the itraconazole NS and marketed injections, with greater concentrations detected in the liver, spleen, and lungs, indicating potential for passive targeting to the mononuclear phagocyte systems [235]. A comparative study evaluated the tissue distribution as well as the pharmacokinetics of two oridonin NCs with markedly different sizes (103.3 ± 1.5 nm and 897.2 ± 14.2 nm) after intravenous delivery in rabbits [236]. It was noticed that the smaller NCs acted similar to a drug solution, while the larger NCs exhibited increased accumulation in the liver, lungs, and spleen. The studies demonstrate that modifying the particle size is crucial for effectively targeting NCs. Smaller crystals target tumor cells by an enhanced permeability and retention effect, while larger crystals are predominantly absorbed by the mononuclear phagocyte system, especially in the spleen and liver. Stabilizers and targeting ligands are non-covalently attached to the surface of NCs, where nonspecific interactions, such as adsorption, play a dominant role during surface modification [28]. Hyaluronic-acid-anchored paclitaxel NCs significantly extended the systemic circulation of the drug, increasing the AUC by 8.4 times compared to the commercial formulation (Taxol™). Additionally, the NCs demonstrated reduced lung metastasis, enhanced antitumor efficacy, and lower toxicity in an LA-7 tumor-bearing rat model compared to Taxol™. In another study, paclitaxel NCs were modified with hyaluronic acid and apo-transferrin (bilobar protein) to evaluate their ability to inhibit cell growth. In MCF-7 cells, the surface-modified NCs achieved 60% cell growth inhibition, though this effect was less potent than that of normal NCs and the pure drug in normal cell lines [237]. The evaluation of Herceptin-functionalized paclitaxel NCs on HER-2-positive breast cancer cells showed that the surface-modified NCs had a good binding affinity and cell-specific uptake in comparison to the drug NCs [238]. Docetaxel NCs stabilized with a chondroitin sulfate A and PEG conjugate, demonstrating key benefits such as pegylation, stabilization, and CD44 receptor targeting [239]. In studies with MDA-MB-231 cells, the drug chondroitin sulfate A NCs exhibited an increased cellular uptake, deeper tissue penetration, and enhanced cytotoxicity, primarily due to the enhanced permeability and retention effect and receptor-mediated endocytosis.

The excessive dilution of the formulation can cause the detachment of stabilizers or targeting ligands. As a result, this shedding poses a significant challenge when the goal is to achieve targeted delivery to a specific site [240]. To reduce stabilizer shedding, strategies include chemically modifying stabilizers to increase the binding sites or using crosslinking techniques to physically entrap the stabilizer around the NCs. A study showed that the layer-by-layer assembly of polyelectrolyte-coated paclitaxel NCs dissolved more slowly compared to uncoated NCs and the commercial formulation (Abraxane^®^) [241]. However, they were rapidly removed from circulation, likely due to the shedding of the PEGylated coating from the surface of the NCs. Even though drug NCs have been rapidly developed for oral and other administration routes, the parenteral NS face a number of challenges including sterility, long-term stability, and translation to clinical use. Currently, only one intravenous NC product, Ryanodex^®^ (dantrolene sodium), is available on the market. Other products, such as meloxicam and ubidecarenone NCs, are in advanced clinical trials.

Clinical trials focus on evaluating safety, efficacy, pharmacokinetics, and optimal dosing across phases, with an emphasis on bioavailability and reduced adverse effects. Patents often cover novel formulation methods, such as advanced manufacturing techniques, surface modifications, and lyophilization processes that enhance stability and dispersibility. Recent advances include the use of SCF technology, targeted delivery systems, sustained release formulations, nanocrystal technology, and smart, stimuli-responsive NS, all of which improve drug solubility, bioavailability, and therapeutic outcomes, particularly in cancer and chronic disease treatments. In summary, NS significantly enhance drug delivery across various administration routes, including oral, pulmonary, ocular, and transdermal. The key features of the most vital NS, their preparation techniques, delivery routes, and applications are summarized in Table 7.

NS offer unique advantages for enhancing the solubility and bioavailability of poorly soluble drugs, featuring simple production processes and broad applications. However, formualtions like liposomes, nanoemulsion, polymeric micelles, solid lipid NPs, and nanostructured lipid carriers excel in targeted delivery and biocompatibility, albeit at higher costs and with stability challenges. Contextualizing NS alongside these alternatives highlights their role as a versatile and cost-effective platform for drug delivery, particularly for hydrophobic drugs (Table 8).

## 6. In Vitro and In Vivo Characterization

**Figure 9:**
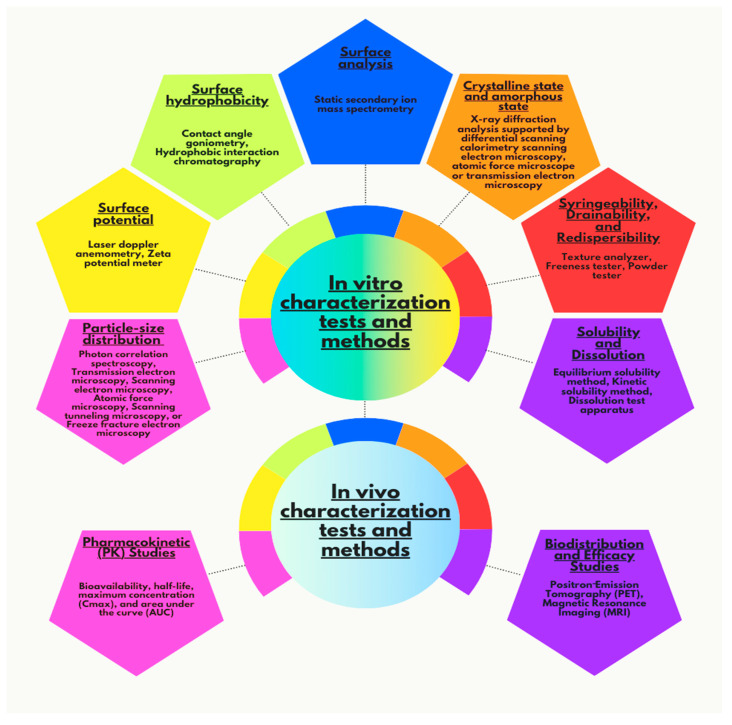
Common physical characterization tests and methods used in nanosuspensions (prepared with BioRender.com, accessed on 25 November 2024).

The in vitro characterization of pharmaceutical NS involves a range of analytical methods to evaluate the critical parameters. The common physical characterization tests and methods used in NS are presented in Figure 9. The contact angle (θ) measurement is essential for assessing the NC wettability in pharmaceutical NS, influencing drug dissolution, bioavailability, and stability. For example, miconazole showed poor wettability with pure water (contact angle >140°), PVP/SDS, and Poloxamer solutions, but demonstrated improved wettability with a 2.5% hydroxypropyl cellulose and 0.1% sodium lauryl sulfate solution (contact angle 43°), aiding in stabilizer selection and optimization [249]. The Flory–Huggins’s interaction parameter (χ), obtained from melting point depression data, evaluates the drug polymer interaction strength and predicts the NS suitability [250]. Higher negative χ values, such as in naproxen polymer systems (HPMC, Soluplus^®^, and Poloxamer), indicate stronger interactions, while smaller values, as observed in budesonide polymer systems, suggest weaker interactions. Combining ‘χ’ with ‘θ’ measurements have proven effective in correlating with successful NS formulation and stability.

Particle size distribution, a key determinant of stability and performance, is assessed using techniques like photon correlation spectroscopy while surface morphology is evaluated using scanning electron microscopy, transmission electron microscopy, scanning tunneling microscopy, or freeze fracture electron microscopy. A wide particle size distribution with a PDI > 0.5 increases the likelihood of Ostwald ripening, which can reduce the drug solubility, dissolution rate, and bioavailability. Therefore, maintaining a narrow particle size distribution (PDI, 0.1–0.25) is essential for ensuring the stability of drug NC suspensions. Surface potential, which impacts colloidal stability, is analyzed using laser doppler anemometry or a ZP meter. An electrostatically stable NC suspension typically requires a ZP of 30 mV. When a polymer stabilizer is used, the ZP on the NC surface, which is primarily influenced by the polymer concentration rather than the surfactant concentration, should be at least 20 mV to ensure stability [251]. Surface hydrophobicity, crucial for understanding wettability and interaction with biological environments, is examined through contact angle goniometry or hydrophobic interaction chromatography. Advanced surface analysis employs static secondary ion mass spectrometry. To distinguish between crystalline and amorphous states, XRD analysis, differential scanning calorimetry (DSC), and microscopic techniques such as scanning electron microscopy, atomic force microscopy, or transmission electron microscopy are utilized. Carvedilol undergoes crystal transformation during freeze-drying but not with wet grinding or spray-drying, as confirmed by P-XRD analysis [38]. While DSC detects melting point changes, it cannot accurately identify polymorphic transitions. FTIR analysis confirmed no interactions between carvedilol and stabilizers like hydroxypropyl cellulose and mannitol. Combining DSC, P-XRD, and FTIR is essential for evaluating crystal transformations and interactions in drug NC development. Suspended-state NMR and Raman spectroscopies are used to assess the molecular states of drugs and stabilizers in NS. The combined measurements revealed the molecular states of indomethacin in NS, with poloxamer 407 adsorbing onto the drug crystal surface [252]. The polypropylene oxide group interacted hydrophobically, while the polyethylene oxide group remained flexible. Wet milling with amorphous indomethacin efficiently produced α-form drug NCs.

Developing an efficient in vitro dissolution method to predict in vivo release is a substantial technical challenge due to the variability and diversity of NC preparations as well as the complexity of release behavior. In vitro release testing by the dialysis sac method effectively differentiates naproxen NC sizes and provides release profiles for various sizes [253]. The dialysis bag method differentiates between the in vitro release behavior of teniposide NS, freeze-dried preparations, and the marketed preparation [254]. In vitro–in vivo correlation is a model that predicts in vivo drug absorption based on in vitro release data, helping optimize drug formulations and reduce clinical testing [255]. Ritonavir NS were prepared through the microfluidization method, and both the in vitro dissolution and in vivo bioavailability were assessed [108]. The formulation enhanced the dissolution and solubility, leading to a positive correlation between in vitro dissolution and in vivo pharmacokinetic parameters. At the early stages of development, the efficient in vitro release testing of crystalline suspensions helps validate the product quality and creates a link with the in vivo performance. Crystalline long-acting parenteral NS were formulated with different stabilizing polymers and tested using the USP-4 dissolution method [256]. The study revealed that salt effects and polymer chain interactions influence the stability and release profiles of polymer-coated NPs, as confirmed by 1D 1H-NMR and 2D NOESY spectroscopy. The findings provide insights into the dynamic interface between nanocrystalline drugs and the aqueous environment during dissolution.

Practical parameters like syringeability and injectability are evaluated using a texture analyzer, while drainability is tested using a freeness tester. Redispersibility is assessed with a powder tester, ensuring ease of handling and reconstitution. Solubility is measured through equilibrium and kinetic solubility methods, and dissolution behavior is studied using standard dissolution test apparatus, providing insights into drug release profiles. Each technique serves specialized purposes depending on the sample type and desired information. Detailed descriptions of various characterization techniques are available in the literature [257].

The in vivo characterization of NS involves pharmacokinetic studies, tissue distribution, efficacy evaluations, and biodistribution assessments. The pharmacokinetic studies assess the absorption, distribution, metabolism, and excretion of a drug, with in vivo bioavailability typically measured by tracking the drug concentration in blood samples over time. In one study with paclitaxel, NS showed an extended plasma circulation time compared to drug injection [247]. They also exhibited passive targeting to mononuclear phagocyte system-related organs like the liver and spleen, potentially improving cancer treatment in these tissues while minimizing side effects in normal tissues. Tissue distribution studies examine how NS distribute throughout the body following administration, often using methods like radiolabeling or fluorescence. In vivo efficacy studies assess therapeutic outcomes, like tumor reduction or anti-inflammatory effects, in animal models. Biodistribution studies employ imaging methods like positron emission tomography or magnetic resonance imaging to provide detailed insights into the in vivo behavior and targeting of NS. Combining in vivo and in vitro methods allows for a comprehensive evaluation, ensuring the NS’s clinical success.

## 7. Marketed Products, Clinical Trials, and Patents

Several commercially available drug products utilize NS technology to enhance drug delivery and therapeutic efficacy for a variety of medical conditions (Table 9). Abraxane^®^ (2005), a paclitaxel NS, addresses the poor solubility of paclitaxel, eliminating the need for solvents like Cremophor EL, which are associated with hypersensitivity reactions. Its approval significantly improved treatment options for metastatic breast cancer and has become widely used in oncology. (1) Invega Sustenna^®^ (2009), a long-acting injectable NS of paliperidone palmitate, provides an extended release formulation for schizophrenia management. Its use reduces the need for daily oral medication, helping to improve adherence in patients with this chronic condition. (2) Triglide^®^ (2005) employs NS technology to enhance the bioavailability of fenofibrate, improving lipid control for patients with hypercholesterolemia compared to traditional formulations. (3) Megace ES^®^ (2005) uses NS technology to improve the absorption of megestrol acetate, a drug used to treat anorexia and cachexia in cancer and HIV/AIDS patients. (4) Cabenuva^®^ (2021), a combination of cabotegravir and rilpivirine NS, offers a long-acting injectable treatment for HIV-1 infection. By reducing the need for daily oral dosing, it represents a significant advancement in HIV care, enhancing convenience and adherence. (5) Market acceptance and regulatory considerations are pivotal in commercializing NS drug products. Regulatory agencies like the FDA and EMA emphasize particle size consistency, stability, safety, and manufacturing reproducibility to ensure product quality. The above examples have gained approval due to their ability to address unmet clinical needs while adhering to these stringent standards. However, higher production costs and competition from other advanced drug delivery systems can pose challenges, making favorable reimbursement policies and strategic pricing crucial. Successful NS products set a precedent for future innovations, fostering confidence in this transformative technology [258,259,260,261,262]. Regulatory considerations play a critical role in the successful commercialization of these NS products. For instance, Abraxane^®^ demonstrated the elimination of toxic excipients, which was a key factor in gaining regulatory approval. Similarly, Invega Sustenna^®^ and Cabenuva^®^ highlighted improved patient compliance and adherence, addressing specific unmet needs in schizophrenia and HIV care, respectively. The success of these products sets a benchmark for future NS innovations, fostering regulatory confidence and paving the way for the broader adoption of NS technology in pharmaceutical development.

Ongoing clinical trials investigate various NS-based drug delivery systems across multiple therapeutic areas (Table 10). Research on BPM31510 examines its safety, pharmacokinetics, and anti-tumor effects in patients with high-grade gliomas, solid tumors, and advanced pancreatic cancer, both alone and in combination with chemotherapy. The potential of ivermectin NS as a nasal spray for treating post COVID-19 anosmia and preventing COVID-19 is also being explored. Trials on drugs like GLPG0555, JNJ-40411813, and rilpivirine compare solid formulations and NS to evaluate absorption, particle size effects, and dietary impacts. Additionally, studies on rilpivirine’s long-acting intramuscular formulations and JNJ-40411813’s effects on sleep, metabolism, and interactions with CYP3A4 inhibitors are underway. These trials highlight the broad applicability of NS technology in enhancing bioavailability, therapeutic efficacy, and patient outcomes in oncology, infectious diseases, and pharmacokinetics. The compilation of recently filed patents for pharmaceutical NS showcases a variety of innovations aimed at improving drug delivery, stability, and therapeutic efficacy across multiple medical conditions (Table 11). These patents emphasize advances in both formulation techniques and the therapeutic benefits of NS, particularly for poorly water-soluble drugs. The patent disclosures collectively point to the growing versatility and application of NS technology across various fields of medicine, from oncology and infectious diseases to inflammation and metabolic disorders. The technology’s ability to improve solubility, stability, and bioavailability, while offering targeted and sustained drug release, holds immense potential for advancing the treatment of complex diseases. Furthermore, innovations in formulation methods, such as reduced stabilizer usage and improved drug targeting mechanisms, suggest a promising future for NS-based drug delivery systems.

## 8. Future Perspectives and Challenges

Advances in pharmaceutical NS are opening new possibilities for targeted drug delivery, with innovations enabling precise particle size and surface modifications to target specific tissues. Integrating targeting ligands, such as antibodies or peptides, or combining them with stimuli-responsive materials could allow for drug release in response to specific triggers, supporting personalized medicine and enhancing precision [263]. NS may also aid in delivering sensitive macromolecules like proteins and nucleic acids, protecting them from degradation, which could expand their use in gene therapy and vaccine delivery [264]. The flexibility of NS allows for administration through oral, parenteral, ocular, pulmonary, and topical routes, with each pathway offering distinct advantages and challenges. Research is ongoing to enhance stability in gastrointestinal environments, reduce aggregation for parenteral routes, and improve ocular retention [113,175]. Innovations in manufacturing methods, such as sonocrystallization and SCF technology, aim to streamline production for greater scalability and cost effectiveness. Emerging technologies like machine learning and artificial intelligence may further refine formulation designs, optimizing parameters for enhanced effectiveness [265].

Dual- or triple-drug NS comprised of natural products hold the potential for synergistic therapies in complex diseases like cancer, where co-formulating multiple drugs could maximize the therapeutic impact while preserving each drug’s stability [220]. Efforts are also underway to establish standardized guidelines for stability, characterization, and toxicity testing to facilitate the transition from research to commercialization. Stability challenges, including particle aggregation, are being addressed through stabilizing agents and lyophilization to extend the shelf life [266]. Future developments in patient-specific NS, potentially guided by genetic profiling, could provide highly individualized therapies. Additionally, theranostic NS, incorporating diagnostic agents, could enable the real-time monitoring of drug distribution and therapeutic responses, integrating both therapeutic and diagnostic capabilities for improved treatment outcomes. The transfer of NS from research laboratories to clinical usage has been expedited by developments in formulation methods and manufacturing procedures. Several intramuscular NS injections are already available, such as Invega Sustenna, aripiprazole lauroxil, and aripiprazole long-acting injections, which are used to treat schizophrenia. Additionally, NS like GSK1265744 and rilpivirine (TMC278) have demonstrated potential for HIV-1 treatment by reaching steady-state plasma concentrations within three days. This trend is expected to grow as pharmaceutical companies explore intramuscular NS due to their benefits, including ease of administration and reduced pain or irritation at the injection sites. These formulations are particularly valuable for patients prone to noncompliance, such as schizophrenia. In preclinical pharmacokinetics studies, NS show promise for immunocompromised patients who take multiple pills daily. Poorly soluble drugs remain good candidates for NS as this formulation can improve pharmacokinetics. Advances in aseptic technologies and the development of functionalized NCs for targeted organ delivery offer potential solutions for future research and commercialization. Recently, NC technology has revolutionized paliperidone as a long-acting antipsychotic, offering improved adherence, efficacy, long-term outcomes, patient satisfaction, safety, and cost effectiveness, establishing it as a significant advancement in psychiatric care [267]. The development of NC-based long-acting medications showcases a promising strategy for drug innovation, with potential applications in managing other chronic diseases. The development of suspension-based long-acting parenteral formulations has increased due to the growing incidence of therapeutic compounds with low aqueous solubility and bioavailability.

Regulatory authorities such as the FDA and EMA emphasize stringent guidelines to ensure the safety, efficacy, and quality of NS. The application of QbD principles, as outlined in ICH Q8 (R2), is a cornerstone of regulatory compliance, involving the identification of CQAs like the particle size and ZP, and critical process parameters such as the milling time and homogenization pressure [268]. Safety concerns include potential toxicity due to enhanced bioavailability and long-term NC accumulation in organs causing toxicity risks, necessitating thorough toxicological evaluations. There is a need for further research on the role of dimensions in the physical, chemical, and biological properties of FDA-regulated nanotechnology-based products. Product-specific premarket reviews, when required, help the FDA understand these products’ characteristics and behavior. For products not subject to a premarket review, the FDA encourages early consultation with the industry to address any regulatory, safety, efficacy, or public health concerns. The FDA will continue to issue additional targeted guidance to support the industry in addressing these issues [269,270]. Furthermore, NS often follow complex regulatory pathways, requiring the detailed documentation of manufacturing and safety data for regulatory submissions [271]. Post-marketing surveillance programs and pharmacovigilance are critical to monitor adverse effects and maintain safety standards.

Artificial intelligence and machine learning have revolutionized pharmaceutical research, particularly in the development of NS, NCs, and other advanced drug delivery systems [272]. Artificial intelligence models, including artificial neural networks, random forests, Monte Carlo simulations, and advanced techniques like genetic algorithms, particle swarm optimization, and the response surface methodology are widely used to optimize formulation parameters, predict stability, and enhance the delivery efficiency. These approaches reduce reliance on trial-and-error experiments, streamline development, and improve accuracy.

Machine learning methods have been effectively applied to predict NC properties prepared through HPH and wet ball milling [265]. Computational techniques, such as Monte Carlo simulations and molecular dynamics, have been employed to minimize experimental repetitions, enhancing efficiency in NC development. Artificial intelligence also aids in scaling up nanocarriers, optimizing compatibility, addressing regulatory compliance, accelerating drug development and improving therapeutic outcomes. Despite challenges such as data requirements and potential biases, artificial intelligence remains a transformative tool in pharmaceutical innovation, particularly for precise and cost-effective NC and NS technologies [273,274,275,276].

## 9. Conclusions

NS are an optimal formulation choice for rigid hydrophobic drugs constrained by their physicochemical properties and dose. NS significantly enhance bioavailability through increased dissolution, solubility, and enable surface modifications for targeted drug delivery. These attributes make them a valuable tool for formulation scientists in addressing challenges associated with diverse drug entities. However, dissolution-based drug delivery is limited to drugs with low solubility and requires the careful control of the particle size and crystal form to ensure a consistent release. The transformation of the crystal form during solidification greatly impacts both the stability and dissolution rate.

Stable crystalline forms ensure long-term drug stability, but metastable or amorphous forms are preferred for class II and IV drugs to enhance bioavailability. Crystallization inhibitors prevent undesirable transformations, while polymeric excipients slow nucleation and growth, unlike hygroscopic excipients that may accelerate transformations. Storage challenges include particle aggregation and settling, necessitating resuspension to ensure accurate dosing. Formulations often rely on additives like wetting agents, viscosity enhancers, and buffers for stability and performance. FDA-approved non-ionic excipients are preferred over ionic surfactants, with natural and self-assembling stabilizers successfully used at lower concentrations to minimize off-target effects in the body. Overcoming challenges related to stability, scalability, regulatory approval, and patient-specific customization will be essential for realizing their full potential. For instance, the long-term stabilization of NCs in polymeric orodispersible films poses a significant formulation challenge, particularly for low-dose drugs and NCs reaching a supersaturated state during dissolution. In such cases, HPMC is a preferable film-forming polymer due to its excellent film-forming properties and ability to stabilize supersaturated drug solutions. To convert liquid NS into dry particulate forms, spray-drying and freeze-drying are the primary techniques, while granulation and HME support efficient downstream processing. Additionally, 3D printing technology offers significant potential for personalized medicine, dose optimization, and the ability to create complex dosage forms.

While NS have garnered significant attention from pharmaceutical researchers, the precise techniques underlying the stabilization, solidification, and dispersibility of dried NS remain inadequately understood. Further investigation is needed to elucidate factors such as the role of stabilizers, drying techniques, particle interactions, and their impact on maintaining the physicochemical properties and therapeutic efficacy of NS during and after processing. Continued research and innovation in this field will likely lead to more effective, safe, and accessible treatments, transforming the landscape of pharmaceutical drug delivery.

## Figures and Tables

**Table 1:** Comparison of various nanosuspension preparation methods and their key characteristics.

Method	Advantages	Limitations	Applications	Scalability	Energy Consumption	Particle Size Uniformity
High-pressure homogenization (HPH)	Excellent particle size control, nonthermal processing	High energy requirements, equipment wear	Oral, parenteral, and ophthalmic delivery systems	High	High	Excellent
Media milling	Low energy consumption, scalability, simple operation	Contamination risk from milling beads, long processing times	Wide range of drugs, including poorly aqueous-soluble ones	Moderate	Moderate	Good
Sonocrystallization	Precise size control, uniform distribution	High energy input, limited scalability	BCS Class II drugs, solubility enhancement	Low	High	Excellent
Supercritical fluid	Minimal solvent use, suitable for thermolabile drugs	High operational costs, specialized equipment	Specialized formulations, environmentally friendly	Moderate	Moderate	Good
Dry co-grinding	Cost-effective, avoids toxic solvents	Potential for agglomeration, not suitable for all drugs	Enhancing solubility and stability	High	Low	Moderate
Emulsion solvent evaporation	Precise particle size control, versatile for different drugs	Requires careful handling of organic solvents	Improving water solubility and bioavailability	Moderate	Moderate	Excellent
Pulsed laser ablation in liquid	High precision, handles heat-sensitive drugs	High costs, complexity, limited scalability	Formulating heat-sensitive drugs, advanced delivery	Low	High	Excellent
Precipitation	Simple, cost-effective, scalable for mass production	Challenges in controlling particle size, risk of agglomeration	Mass production, nanoparticle (NP) stabilization	High	Low	Good

**Table 2:** Selected examples of oral nanosuspensions with active(s), stabilizer(s), method of preparation, and key features.

Drug	Stabilizer	Technique	Highlights	Reference
5-fluorouracil	Soy protein	Solid dispersion via kneading method followed by suspension in sodium alginate vehicle	Soy protein–fluorouracil and soy protein carrageenan kappa-fluorouracil solid dispersions were prepared, characterized, and suspended in a sodium alginate gel-forming vehicle with excess soy protein. The optimal formulations reduced drug release by over 48% compared to the drug alone, as NPs were released as alginate-coated particles. Studies on colorectal cancer cell lines by F5 NPs showed enhanced time-dependent efficacy than free drug.	[112]
Curcumin	Polyethylene glycol (PEG)	Emulsion-templated freeze-drying	The optimized nanosuspension (NS) has nano particle size (211 nm), relatively low polydispersity index (PDI, 0.06), and good zeta potential (ZP) of −25 mV. By varying active agent and excipient concentrations, as well as sonication time, the size and PDI were fine tuned. In vitro bioaccessibility tests revealed increased curcumin dissolution, although this was accompanied by reduced stability compared to unformulated curcumin. Cytotoxicity studies showed the NS had a lower toxicity profile compared to solubilized curcumin.	[113]
Curcumin	Hydroxypropyl methylcellulose (HPMC)	Antisolvent precipitation	Freeze-dried NS with a particle size of 193.5 nm and PDI of 0.261 were encapsulated into microbeads via ionotropic gelation technique using chitosan and pectin as rate-controlling polymers. The optimized microbeads showed 89.45% entrapment efficiency and 14.54% drug loading, with colon-specific drug release. Pharmacokinetic studies in Wistar rats revealed that microbeads demonstrated higher Cmax (2.5-fold) and AUC_0–48_ (4.4-fold) than plain curcumin due to reduction in particle size.	[114]
Cyclosporine A	HPMC and sodium dodecyl sulfate (SDS)	Wet milling	Optimized NS had favorable particle size (600 nm), PDI (0.4), and ZP (-25 mV). Its solubility was 4.5 times higher and its dissolution rate exceeded that of the commercial formulation. AUC_0–24_ values for the NS were 2.09 (fasting) and 5.51 times (Fed) greater than the powder. Following 21 days of oral dosing, immunological tests showed that the NS group’s CD4+/CD8+ ratio remained superior to that of the commercial product.	[115]
Rutin	HPMC E15, Soya lecithin and SDS	Antisolvent precipitation	Optimized NS has an average size of ~122.85 nm, higher dissolution rate (87.63%), and AUC_0–24_ (3-fold) than the rutin (39.77%). In MG-63 osteoblast cells, it enhanced cell growth, antioxidant capacity, and osteocalcin synthesis. Enhanced biochemical markers and bone condition confirmed the protective effect of developed NS, suggesting its potential as an effective therapy for bone health.	[116]
Simvastatin, glimepiride	Sodium lauryl sulfate and PVP K-30	Antisolvent precipitation followed by spray-drying	NS and self-nanoemulsifying drug delivery systems of glimepiride and simvastatin resulted in 6.4-fold and 4.45-fold increase in dissolution rates and drug permeability, respectively. Pharmacokinetics data in rats showed improvement in oral bioavailability with a 6.69- and 2.68-fold increase for glimepiride and simvastatin, compared to their pure form. Both dissolution rates and bioavailability were significantly improved with the NS.	[117]

**Table 3:** Conversion of oral nanosuspensions; types, advantages, limitations, preparation methods, and typical applications.

Formulation Type	Advantages	Limitations	Preparation Techniques	Applications
Capsules	Convenient dosing, high drug-load capacity	Limited to drugs stable in capsule form	Encapsulation using liquid or powder NS	Oral administration of NS, targeted delivery
Film	Enhanced drug absorption, flexibility in design, adaptable for patient-specific formulations	Moisture sensitivity, requires precise manufacturing, limited scalability for specialized techniques	Casting, solvent evaporation, electrospinning, 3D printing	Dissolution enhancement, rapid or sustained drug release, personalized medicine
Pellets	Improved bioavailability, consistent release profile	Complex preparation process, expensive equipment	Extrusion spheronization, spray granulation	Multiparticulate drug delivery, modified release systems
Powders	Enhanced stability, easy reconstitution	Risk of aggregation, requires redispersants	Lyophilization, spray-drying, fluidized bed drying	Reconstitutable suspensions, pediatric and geriatric formulations
Tablets	Controlled drug release, ease of administration	May require coating for controlled release	Direct compression, wet granulation	Solid oral dosage forms, controlled drug delivery

**Table 4:** A summary of different pharmaceutical nanosuspension preparations developed for various ocular disorders and their characteristics.

Active (s)	Stabilizers	Method	Indication	Highlights	Reference
Cyclosporine A	D-alpha-tocopheryl polyethylene glycol 1000 succinate (TPGS)	Antisolvent precipitation	Dry eye disease	Nanoscale drug formulation (NS@lipid-PEG/CKC) coated with PEGylated phospholipids demonstrated enhanced ocular penetration and cellular uptake. NPs, ~173 nm in size and with ZP of +40 mV, showed efficient mucus permeation, biocompatibility, improved cellular uptake, and extended precorneal retention without causing ocular irritation. In a rat model of dry eye disease, this formulation significantly improved tear production and goblet cell density compared to a commercial cationic nanoemulsion.	[159]
Deferasirox	Polyvinyl alcohol (PVA)	Wet media milling	Age-related macular degeneration	NS were loaded into polymeric dissolving ocular microneedles, which demonstrated high solubility compared to pure drug, along with enhanced thermal and short-term stability. They exhibited effective scleral penetration, with optical coherence tomography images indicating an 81.23 ± 7.35% insertion depth into full-thickness porcine sclera. Drug deposition studies showed 64% scleral drug retention within 5 min, nearly five times greater than with pure-drug-loaded microneedles. In vitro cell viability testing on human retinal pigment epithelial cells demonstrated the safety and suitability of this formulation for ocular use.	[160]
Ganciclovir	PVA and sodium lauryl sulfate	Acid–base nanoprecipitation combined with ultrasonication	Human cytomegalovirus retinitis	Detachable dissolving microneedles (DMN) system loaded with prepared NS exhibited robust mechanical strength. The permeation of the drug from microneedles across porcine scleral tissue was higher than the drug powder and drug NS (*p* < 0.05). A higher percentage of drug accumulated in the scleral tissue, as the embedded needles served as a drug reservoir, gradually released to the target site for up to 7 days.	[161]
Ganciclovir	Eudragit RL100	Nanoprecipitation	Herpes simplex virus keratitis	Prepared NPs exhibited ideal particle size (260.8 nm), entrapment efficiency (51.06%), ZP (−10 mV), and PDI (0.158) for ocular delivery. Microscopy images demonstrated that the NPs possess spherical shape and smooth surfaces. Optimized NPs demonstrated a sustained release profile and improved corneal permeation compared to the drug solution.	[162]
Prednisolone acetate	Mannitol, PVA, and TPGS	Spray-drying	Post-operative surgery, allergies, and swelling	Optimized composition of drug: mannitol: PVA (0.3:0.67:0.03%) demonstrated physical stability for 3 months when stored at 40 °C/75% RH. The NS achieved a crystal size of 590 ± 165 nm, osmolality of 297 ± 6 mOsm/L, and viscosity of 11 ± 8 cP, with rapid and complete drug dissolution in 120 s in 1% surfactant media. In ex vivo goat studies, corneal adhesion was 6.2 times higher than that of control (microsuspension). Safety evaluations using corneal histopathology and hen egg test chorioallantoic membrane assay indicated no adverse effects on the cornea or capillary integrity.	[163]
Triamcinolone acetonide	Sodium deoxycholate, and soluplus	Nanoprecipitation using the solvent–antisolvent method	Posterior uveitis	Optimized drug NCs had nano size (~243 nm) and good yield (~90%). Ocular pharmacokinetic studies using in situ gel of NCs demonstrated a higher peak concentration (Cmax = 854.9 ng/mL) and prolonged drug presence (MRT_0–∞_ = 11.2 h) in the vitreous humor compared to aqueous suspension of the drug with 20% hydroxypropyl β-cyclodextrin.	[164]

**Table 5:** Description of nanosuspension formulations used in transdermal delivery.

Active (s)	Method	Stabilizer	Highlights	Reference
Cannabidiol	Antisolvent precipitation	Tween 80	Developed NS demonstrated small particle size (166.83 ± 3.33 nm), favorable PDI (0.21 ± 0.07), and higher concentration (46.11 ± 0.52 mg/mL). Optimized DMN, fabricated with 11.6% (w/w) hyaluronic acid, 5% (w/w) PVP K30, and 0.4% (w/w) trehalose, and loaded with drug NS. This formulation showed good intradermal dissolution and mechanical strength, enhanced skin delivery, prolonged in vivo effect, and improved bioavailability. In a rat model of knee synovitis, the product demonstrated better therapeutic efficacy and safety than subcutaneous methotrexate injections, significantly reducing TNF-α and IL-1β levels.	[191]
Centella Asiatica	Wet bead milling	PVP K30	Optimized NS system, consisting of 10% (*w/v*) extract, 0.5% PVP K30, and 89.5% distilled water, was spherical or elliptical, about 200 nm in size, and exhibited low crystallinity. Dissolution of active constituents from the NS was significantly faster than pure drug. The high payload NS formula notably increased skin penetration of active constituents in porcine skin, compared to the marketed Madeca cream (1% Centella Asiatica). Additionally, topical application of the NS system was well tolerated in rats, showing no signs of erythema or edema.	[192]
Deferasirox	Wet bead milling	PVA	When compared to the pure drug, NS release demonstrated a threefold spike in dissolution rate. Optical coherence tomography verified an implantation depth of 378 µm in pig skin. Mechanical testing on NS-loaded DMNs indicated sufficient strength for effective skin penetration. Studies on skin deposition revealed that the NS DMN system delivered 60% of the medication, which was significantly higher than the transdermal patch (which does not involve needles) or pure drug DMNs.	[193]
Luliconazole	Homogenization with high-speed magnetic stirring and probe sonication	Esterified starch polymer	NS with sizes of 369.1 to 745.4 nm and favorable properties (PDI; 0.193–0.344, ZP; 22–45 mV) were effective in enhancing drug loading within micelles. Molecular docking showed strong binding between the drug and the polymer. When incorporated into a Carbopol 934 gel, the NS had good drug content, spreadability, pH, and viscosity. The nanogel improved drug skin permeation (83.818 μg/cm^2^) compared to a standard cream (70.085 μg/cm^2^) and increased drug accumulation in skin cells threefold.	[194]
Oxybutynin	Solvent–antisolvent precipitation and high-pressure homogenization	Poloxamer 407 and Vitamin E	Ex vivo and in vivo studies revealed that drug nanogel considerably improved transdermal delivery, achieving 4-fold (permeation) and 3-fold (pharmacokinetics) increases compared to suspension, respectively. In vitro retention tests showed that nanogel increased drug concentration in the epidermal and dermal area by 3-fold than suspension gel. It utilizes hair follicle pathways, improves drug retention in the skin, and has no skin irritation.	[195]
Rosuvastatin calcium	Precipitation–ultrasonication	Propylene glycol and Tween 80	An optimized transdermal patch (P4) exhibited higher drug permeation (86.01%), along with superior thickness (0.86), weight uniformity (475 mg), folding endurance (279.3), moisture uptake (7.06%), and moisture content (6.81%). Additionally, the formula demonstrated excellent physical and chemical stability.	[196]
Rotigotine	Wet bead milling	PVA	NS showed low particle size (274.09 ± 7.43 nm), PDI (0.17 ± 0.04), and ZP (−15.24 ± 2.86 mV). NS showed a greater dissolution rate than drug powder. Drug-loaded dissolving microarray patch, consisting of a drug layer and a drug-free baseplate, delivered significantly more drug per unit area (~0.52 mg/0.5 cm^2^) than the conventional transdermal patch, Neupro^®^ (~0.20 mg/1 cm^2^) in porcine skin. Pharmacokinetic studies confirmed that despite being smaller in size (2 cm^2^ for microarray patch vs. 6 cm^2^ for Neupro^®^), the former achieved comparable drug levels.	[197]

**Table 6:** Nanosuspensions used in pulmonary drug delivery and their highlights.

Active (s)	Method	Stabilizer	Highlights	Reference
Ciprofloxacin with N-acetylcysteine	Antisolvent precipitation technology followed by ultrasonication and spray-drying	HPMC E5	A pulmonary inhalation powder NS was developed with ciprofloxacin and N-acetylcysteine. The aerosol performance tested with the twin-stage impinger and next-generation impactor indicates an impressive fine particle fraction ranging from 68.93% to 77.75%. Co-crystallization with N-acetylcysteine improved the solubility of ciprofloxacin, while L-leucine enhanced the aerosol properties of the dry powder. This formulation demonstrated strong antibacterial activity at lower doses, potentially reducing the risk of systemic toxicity from higher doses.	[214]
Coenzyme Q10	High shear homogenization combined with HPH	Lecithin, PEG32 stearate, and TPGS	Coenzyme Q10 NS, with particle sizes under 100 nm, exhibit good aerosolization properties and low cytotoxicity on A549 human lung adenocarcinoma cells. Nebulized formulations stabilized with PEG32 stearate or lecithin experience particle size growth over time, which impacts nebulization and reduces the antioxidant dose delivered. In contrast, formulations containing TPGS remain stable for 3 months without affecting nebulization performance. The PEG32 stearate NS had highest respirable fraction (70.6%) and smallest mass median aerodynamic diameter (3.02 µm).	[215]
Curcumin and beclomethasone dipropionate	Wet ball media milling	Poloxamer 188	Individual (curcumin and beclomethasone) NS and a multicomponent formulation containing both drugs showed uniformly sized nanocrystals with a mean diameter of 200–240 nm. Multicomponent NS was found stable for 90 days at 25 °C without aggregation or sedimentation. Nebulization tests with the next-generation impactor revealed optimal aerodynamic properties with a mass median aerodynamic diameter below 5 µm.	[216]
Fluticasone propionate	Wet milling combined with HPH	Cholesterol and TPGS	NS with particle size of 246 ± 2.94 nm, administered intratracheally, improved mucociliary clearance, and extended local anti-inflammatory effect. Phospholipid-coated fluticasone NS with a particle size of 192 ± 3.87 nm, delayed drug dissolution, increasing the AUC in lung tissues by 1.72-fold compared to a conventional NS, but decreased pharmacological efficacy.	[217]
Resveratrol	HPH followed by spray-drying	SDS, sodium alginate, chitosan, and PVA	The type of stabilizer, including PVP (1%), significantly impacted the lung morphology, deposition, and drug release of the NS, achieving the highest fine particle fraction. Increasing the stabilizer concentration affected the NS morphology and flowability, leading to lower drug mass in aerosol. The use of stabilizers enhanced drug retention and reduced systemic drug exposure.	[218]
Silybin	Spray-drying	Tween 20 and poloxamer 188	A rat model of bleomycin-induced idiopathic pulmonary fibrosis was employed to evaluate the effects of inhaled silybin from NS. The findings revealed that the inhaled silybin powder reduced inflammation and fibrosis, restricted hydroxyproline buildup in the lungs, regulated gene expression involved in fibrosis progression, and enhanced postoperative survival.	[219]

**Table 7:** Principal features of selected vital nanosuspensions: key attributes, preparation techniques, delivery routes, and applications.

Drug	Key Features	Nanosuspension Technique	Route	Applications	References
Aceclofenac	Enhances dissolution rate and bioavailability for BCS Class II drugs, reduces GI effects	Antisolvent precipitation	Transdermal	Pain management and treatment of inflammatory conditions	[242]
Aprepitant	Overcomes solubility and permeability issues for BCS Class IV drugs, enhances local concentration gradient	Wet media milling	Oral	Chemotherapy-induced nausea and vomiting, postoperative nausea management	[243]
Curcumin	Enhances bioavailability and therapeutic efficacy by improving solubility of this natural antioxidant	HPH technique and probe sonicator	Brain	Anti-inflammatory, cancer therapy, neurodegenerative disease treatment, and multiple biological activities	[111]
Cyclosporine A	Improves oral bioavailability and stability, addressing extensive first-pass metabolism	Wet media milling	Oral	Organ transplantation, autoimmune disorders like rheumatoid arthritis and psoriasis	[115]
Everolimus	Improves solubility and bioavailability	Injection	Ocular	Organ transplantation, autoimmune disorders	[244]
Griseofulvin	Enhances dissolution-limited absorption for BCS Class II drugs by increasing surface area	Wet media milling	Subcutaneous	Treatment of fungal infections like dermatophytosis, tinea capitis, and onychomycosis	[245]
Ibuprofen	Maintains solubility across varying pH environments, stabilizing it in supersaturated states	Antisolvent crystallization	Oral	Pain relief, fever reduction, anti-inflammatory therapy	[246]
Itraconazole	Enhances solubility for antifungal activity	Solvent evaporation	Ocular	Treatment of fungal infection	[167]
Paclitaxel	Improves solubility and sustained release behavior	Microprecipitation—HPH	Oral	Cancer therapy, including breast and ovarian cancers	[247]
Rifampicin	Enhances pulmonary delivery and therapeutic efficacy in tuberculosis treatment	Miniaturized wet bead milling	Pulmonary	Treatment of tuberculosis with enhanced lung deposition	[248]

**Table 8:** Comparative analysis of nanosuspensions and alternative nanotechnologies.

Parameter	Nanosuspensions	Nanoemulsions	Liposomes	Polymeric Micelles	Solid Lipid Nanoparticles/Nanostructured Lipid Carriers
Solubility enhancement	Excellent: effective for hydrophobic drugs	Excellent: improves solubility of poorly soluble drugs	Moderate: suitable for hydrophilic and lipophilic drugs	Good: enhances solubility of hydrophobic drugs	Moderate: improves solubility and protection of labile drugs
Targeted delivery	Limited: primarily enhances solubility, not site-specific delivery	Good: enables delivery to specific tissues	Excellent: enables site-specific drug delivery	Limited: not inherently site-specific	Good: site-specific potential
Stability	Moderate: aggregation risk during storage	Good: thermodynamically stable under appropriate conditions	Low: prone to leakage and instability	Low: rapid dissociation in vivo	Good: solid core provides better stability
Cost of production	Low: simple composition and process	Moderate: requires specialized emulsification techniques	High: complex manufacturing processes	Moderate: relatively simple self-assembly	Moderate: costs vary with lipid source
Scalability	Excellent: easily scalable using homogenization or milling techniques	Moderate: requires high-pressure homogenizers	Moderate: requires specialized equipment	Good: scalable using polymeric systems	Good: scalable using standard lipid processing methods
Biocompatibility	Good: minimal excipients improve compatibility	Excellent: biocompatible with natural oils and surfactants	Excellent: natural phospholipid components enhance compatibility	Good: amphiphilic polymers are generally biocompatible	Good: biodegradable lipids ensure safety
Encapsulation efficiency	Moderate: limited by stabilizer properties	High: suitable for hydrophobic and hydrophilic drugs	High: capable of dual encapsulation	High: excellent for small, hydrophobic drugs	Moderate: limited by lipid matrix capacity
Drug-loading capacity	High: suitable for high drug loads	Moderate: dependent on oil phase and emulsifiers	Moderate: limited by bilayer structure	Moderate: limited by micelle size and stability	Moderate: suitable for lipophilic drugs
Applications	Broad: oral, ocular, parenteral, and pulmonary delivery	Versatile: oral, ocular, and dermal delivery	Moderate: parenteral and targeted cancer and gene therapy	Moderate: hydrophobic drug delivery and cancer therapy	Broad: oral, topical targeted delivery, gene therapy, and parenteral

**Table 9:** Marketed formulations of nanosuspensions.

Product	Drug Compound	Indication	Company	Route	Year
Vitoss^®^	Calcium phosphate	Bone substitute	Stryker	Intraosseous	2001
Avinza^®^	Morphine sulfate	Pain	King Pharmaceuticals	Oral	2002
Ritalin^®^	Methylphenidate	Psychostimulant	Novartis	Oral	2002
Herbesser^®^	Diltiazem	Hypertension	Mitsubishi Tanabe	Oral	2002
Emend^®^	Aprepitant	Antiemetic	Abraxia Biosciences	Oral/Parenteral	2003
Focalin^®^XR	Dexmethylphenidate	Psychostimulant	Novartis	Oral	2005
Triglide^®^	Fenofibrate	Hypercholesterolemia	First Horizon Pharma	Oral	2005
Megace ES^®^	Megestrol acetate	Anti-anorexic	Par Pharmaceutical Companies	Oral	2005
Invega Sustenna^®^	Paliperidone palmitate	Schizophrenia	Johnson & Johnson	Intramuscular	2005
Abraxane^®^	Paclitaxel	Metastatic breast cancer	Merck	Parenteral	2005
Naprelan^®^	Naproxen	Pain	Pfizer/Wyeth	Oral	2006
NanOss^®^	Hydroxyapatite	Bone substitute	Rti Surgical	Intraosseous	2007
Besivance^®^	Besifloxacin	Ocular infection	Bausch & Lomb Inc.	Ocular	2009
Tobradex ST^®^	Tobramycin	Ocular inflammation and infection	Santen Pharmaceuticals	Ocular	2009
Ryanodex^®^	Dantrolene	Malignant hyperthermia	Eagle Pharmaceuticals	Parenteral	2014
Inveltys^®^	Loteprednol etabonate	Postoperative ocular inflammation and pain	Kala Pharmaceuticals	Ocular	2018
Verkazia^®^	Loteprednol etabonate	Dry eye	Santen Pharmaceuticals	Ocular	2018
Cabenuva^®^	Cabotegravir/rilpivirine	HIV-1 infection	ViiV Healthcare	Intramuscular	2022

**Table 10:** Various interventional clinical trials using formulations based on nanosuspensions (https://clinicaltrials.gov/; accessed on 21 November 2024).

NCT Number	Clinical Trial	Study Title	Potential Outcomes	Interventions	Phases
NCT03020602	To investigate the side effects and optimal dosage of ubidecarenone injectable NS (BPM31510) in the treatment of patients with recurrent high-grade gliomas (glioblastoma or anaplastic astrocytoma) who have received bevacizumab treatment. By inhibiting some of the enzymes required for cell growth, BPM31510 may prevent the growth of malignant cells.	BPM31510 in treating patients with recurrent high-grade glioma previously treated with bevacizumab	Gliosarcoma, recurrent glioblastoma, astrocytoma of the brain, glioblastoma	Laboratory biomarker analysis, pharmacological study, ubidecarenone injectable NS	Phase 1
NCT04951362	To study the expected effect of ivermectin NS as nasal spray upon post-COVID-19 persistent anosmia	Role of ivermectin NS as nasal spray in treatment of persistent post-COVID-19 anosmia	Anosmia	Intranasal spray ivermectin	Phase 2, phase 3
NCT01278095	To compare the pharmacokinetics of a solid capsule formulation of GLPG0555 with an NS, and to assess its safety and tolerability of a single dose	Oral bioavailability of GLPG0555 in different solid formulations	Pharmacokinetics comparison of solid capsule GLPG0555 and NS formulation	GLPG0555 solid dispersion, GLPG0555 NS	Phase 1
NCT02650804	To investigate the safety and efficacy of BPM31510 given as a second- or third-line treatment for advanced pancreatic cancer patients over 144 h (two doses of 110 mg/kg given over 72 h); continuous intravenous infusion in conjunction with gemcitabine	BPM31510 administered intravenously with gemcitabine in advanced pancreatic cancer patients	Pancreatic cancer	BPM31510 NS injection, gemcitabine	Phase 2
NCT03002935	To evaluate the pharmacokinetics, pharmacodynamics, and safety of BPM31510 taken orally in healthy individuals	A safety study of orally administered BPM31510 in healthy subjects	Pharmacokinetics, dynamics, and safety evaluation of BPM31510	BPM31510 oral NS 4%	Phase 1
NCT01957735	To investigate the dose-limiting toxicities of BPM31510 in patients with solid tumors when given as a continuous intravenous infusion for 144 h as monotherapy (treatment Arm 1) and in conjunction with chemotherapy (treatment Arm 2)	BP31510 (ubidecarenone, USP) NS for intravenous injection to patients with solid tumors	Metastatic cancer, cancer, solid tumor	BP31510 monotherapy, BP31510 in combination with chemotherapy	Phase 1
NCT04716569	To examine the possibility of ivermectin mucoadhesive NS as a preventative and early management approach for COVID-19	Evaluation of ivermectin mucoadhesive NS as a nasal spray in the management of early COVID-19	COVID-19	Intranasal ivermectin spray	Phase 2, Phase 3
NCT02547870	This study compares the single-dose pharmacokinetics of rilpivirine (RPV) in healthy adult subjects following intramuscular injection of RPV-LA (rilpivirine long-acting parenteral formulation) versus ’aged’ RPV-LA	A study to evaluate the pharmacokinetic effects of different storage conditions for a long-acting NS of rilpivirine on pharmacokinetics	Human immunodeficiency virus type 1	Rilpivirine, RPV-LA, aged RPV-LA	Phase 1
NCT01932320	This study is conducted in 3 parts. Part 1, compares the rate and extent of absorption of a single dose of two solid dose formulations relative to the NS formulation of JNJ-40411813. Part 2 evaluated the effect of a high-fat/high-calorie breakfast on the rate and extent of absorption of the selected JNJ-40411813. Part 3 explores the influence of a potent inhibitor of CYP3A4, ketoconazole, on the rate and extent of absorption of the selected JNJ-40411813.	A study to evaluate the relative bioavailability, food effect and effect of ketoconazole on the rate and extent of absorption of solid dosage formulation(s) of JNJ-40411813	Comparison of absorption and CYP3A4 inhibition of solid and NS JNJ-40411813 formulations	JNJ-40411813 formulation and ketoconazole	Phase 1
NCT03127189	This study’s primary goal is to describe the single-dose pharmacokinetics of rilpivirine in adult subjects following intramuscular injection of rilpivirine long-acting parenteral NS with varying particle size distribution	A study to investigate the effect of different particle sizes on the single-dose pharmacokinetics of rilpivirine of a long-acting NS	Comparison of pharmacokinetics of rilpivirine in varying particle size distribution	Rilpivirine and rilpivirine long-acting parenteral	Phase 1
NCT01951053	The study’s goal is to assess JNJ-40411813’s effects on deep sleep and rapid eye movement sleep as well as its pharmacokinetics, safety, and tolerability in males	A study to evaluate the safety, tolerability, pharmacodynamics, and pharmacokinetics of JNJ-404118413	Assessment of pharmacokinetics, deep sleep, and rapid eye movement of JNJ-40411813	JNJ-40411813, placebo, and citalopram	Phase 1
NCT02740985	To evaluate the pharmacokinetics, safety, tolerability, and early anti-tumor effectiveness of increasing dosages of AZD4635 in patients with advanced solid cancers, both alone and in combination	A phase 1 clinical study of AZD4635 in patients with advanced solid malignancies	Advanced solid malignancies, non-small-cell lung cancer, metastatic castrate-resistant prostate carcinoma, colorectal carcinoma	AZD4635, durvalumab, abiraterone acetate, enzalutamide, oleclumab, docetaxel	Phase 1

**Table 11:** List of pharmaceutical nanosuspensions of most recent patent applications.

Application ID	Date of Publication	Title	Summary of Invention	Potential Benefits
US 20240285545 A1	29 August 2024	Nanosuspension formulation for treatment of pulmonary fibrosis	An NC-based suspension of nintedanib was created through wet milling with Pluronic^®^ F127. The nonadhesive nature of the formulation reduces interactions with barriers that could otherwise deactivate the therapy in the lung.	Pulmonary fibrosis
US 20210361571 A1	25 November 2021	Nanosuspension of cannabidiol for developing water-dispersible formulations	Aqueous NS of cannabidiol with enhanced stability and shelf life have been developed. These NS, which use ethyl maltol as an emulsifier, can be prepared via melt emulsification or solvent evaporation.	Additives in beverages
US 20210315831 A1	14 October 2021	Preparation of nanosuspension comprising nanocrystals of active pharmaceutical ingredients with little or no stabilizing agents	This method describes a technique to produce a nanostructured powder of crystalline agglomerates by mixing drug solution with an antisolvent and evaporating the mixture. The technique utilizes a low ratio of stabilizer to drug.	Nanocrystal development
US 11058635 B1	13 July 2021	Oral administration of fluorouracil in a gelling nanosuspension for targeted delivery to treat colorectal cancers	A sustained release fluorouracil NS, coated with sodium alginate and carrageenan, targets the colon for controlled drug release. The formulation forms a gel in the stomach, which dissolves in the small intestine, ensuring efficient drug delivery to the colon, where it acts on cancer cells.	Colorectal cancer
US 20200390737 A1	17 December 2020	Nanosuspension of salsalate and methods of using the same	The invention describes NS of salsalate or its salt, with particle sizes under 1 micron, combined with a surfactant	Anti-inflammatory and analgesic
US 20200246278 A1	6 August 2020	Injectable diethylstilbestrol nanosuspension formulation	Formulations with diethylstilbestrol-loaded particles in an NS for subcutaneous delivery, providing sustained release while reducing first-pass metabolism and hepatic exposure	Prostate cancer
US 20200129533 A1	30 April 2020	Pharmaceutical nanosuspension for the therapy of HIV infection	A pharmaceutical NS comprised of a crystalline or polycrystalline form of the active ingredient, as long-acting injectable in the long-term supportive therapy of HIV/AIDS	HIV/AIDS
US 20190015349 A1	17 January 2019	Mucus-penetrating budesonide nanosuspension enema for local treatment of inflammatory bowel disease	A budesonide NS with a Pluronic F127 coating enhances penetration of colorectal mucus and ulcerated tissues, improving local delivery for treating colorectal inflammation	Inflammatory bowel disease
US 20180250227 A1	6 September 2018	Nanosuspension formulation	Pharmaceutical or nutraceutical dosage form prepared using a NS comprised of a poorly soluble pharmaceutical or nutraceutical active ingredient, sodium or potassium alginate, and water	Pharmaceutical/nutraceutical dosage

## Data Availability

The data presented in this study are contained within this article.
